# pH-Responsive Chitosan–Alginate Hydrogel Beads Co-Encapsulating Jujube Polysaccharides and cAMP: Enhanced Antioxidant Activity and Gastrointestinal Controlled Release

**DOI:** 10.3390/gels12100893

**Published:** 2026-10-02

**Authors:** Guanghui Zhao, Chi Han, Weihua Wang, Jiankang Lu, Chunlan Zhang, Khan Khalid Nazeer, Xinxin Huang, Kozykan Sabira

**Affiliations:** 1 College of Food Science and Engineering, Tarim University, Alar 843300, China; 15569191897@163.com (G.Z.);; 2 Production & Construction Group Key Laboratory of Special Agricultural Products Further Processing in Southern Xinjiang, Alar 843300, China; 3 College of Information Engineering, Minzu University of China, Beijing 100081, China; 4 College of Modern Agriculture and Food Engineering, Xinjiang Vocational University of Technology, Caohu 844004, China; 5 Modern Agricultural Engineering Key Laboratory, Department of Xinjiang Uygur Autonomous Region, Universities of Education, Caohu 844004, China; 6 Faculty of Engineering Technology, Kazakh National Agrarian Research University, Almaty 050010, Kazakhstan

**Keywords:** biopolymers, jujube polysaccharides, cyclic adenosine monophosphate, chitosan-alginate hydrogel beads, co-encapsulation, pH-responsive release, enhanced antioxidant activity

## Abstract

Jujube polysaccharides (ZJPA) and cyclic adenosine monophosphate (cAMP) are bioactive compounds with antioxidant and health-promoting potential, and their combination exhibits enhanced antioxidant activity. However, their limited stability and susceptibility to gastrointestinal environmental stresses restrict their applications in functional foods. In this study, a pH-responsive composite hydrogel bead delivery system was developed using chitosan (CS) and sodium alginate (SA) as wall materials to co-encapsulate ZJPA and cAMP, aiming to protect these bioactive compounds during gastrointestinal digestion while maintaining their enhanced functionality. The hydrogel beads were fabricated via the ionotropic gelation method, and the formulation parameters were optimized using single-factor experiments combined with response surface methodology (RSM). The optimized hydrogel beads were obtained under the conditions of calcium chloride concentration of 3%, CS concentration of 0.5%, SA concentration of 3%, and core-to-wall ratio of 1:6, with a cAMP microencapsulation yield (MEY) of 97.79%. Structural characterization confirmed that ZJPA and cAMP were successfully incorporated into the CS-SA network through electrostatic interactions and intermolecular hydrogen bonding without the formation of new covalent bonds. Furthermore, the hydrogel beads exhibited distinct pH-responsive swelling and gastrointestinal release behaviors, providing effective protection under acidic gastric conditions and enabling sustained release under intestinal conditions. Importantly, the DPPH radical scavenging assay demonstrated that the co-encapsulation strategy preserved the enhanced antioxidant activity between ZJPA and cAMP. Overall, this study establishes a multifunctional hydrogel bead delivery strategy integrating enhanced bioactivity preservation, gastrointestinal protection, and controlled release, providing new opportunities for the application of jujube-derived bioactive compounds in functional foods.

## 1. Introduction

Jujube (*Ziziphus jujuba* Mill.), belonging to the family Rhamnaceae, is an important medicinal and edible resource widely consumed in China [1]. Particularly in regions like Xinjiang, the jujube industry has rapidly developed into a prominent agricultural sector due to its vast cultivation scale and production output [2]. Jujube fruits are rich in diverse bioactive phytochemicals, notably polysaccharides, triterpene acids, phenolic compounds, and cyclic nucleotides [3]. Among these, jujube polysaccharides (ZJPA) and cyclic adenosine monophosphate (cAMP) represent two of the most prominent functional constituents. ZJPA accounts for approximately 1.31% to 2.22% of jujube dry matter, constituting a major structural and functional component of the fruit [4]. Extensive investigations have confirmed that ZJPA exhibits remarkable biological activities, including antioxidant, immunomodulatory, and gastrointestinal protective effects, showing tremendous potential for nutritional product development [5]. Recent studies have continued to explore and validate these functionalities. For instance, research has focused on the fingerprinting evaluation of jujube polysaccharides and their regulatory effects on gut microbiota, highlighting their role in maintaining intestinal health. Furthermore, advancements in extraction technologies, such as ultrasound-assisted enzymatic methods, have been investigated to improve the yield and antioxidant activity of ZJPA. The potential of ZJPA as a functional ingredient has also been demonstrated in microencapsulation systems, where it was used to enhance the survival of probiotic bacteria during simulated gastrointestinal digestion. Concurrently, jujube is recognized as one of the richest natural plant sources of cAMP—yielding 100 to 600 nmol/g—which is a vital nucleotide derivative well-known for its broad physiological regulatory functions [6].

Despite their remarkable bioactivities, the practical application of ZJPA and cAMP in functional foods is severely hindered by their susceptibility to environmental stressors and gastrointestinal degradation. Specifically, cAMP is highly vulnerable to acidic cleavage in the stomach, whereas the structural integrity and bioactivity of ZJPA can be compromised during food processing, storage, and gastrointestinal transit. Traditional delivery strategies have predominantly focused on single-component encapsulation, which often fails to address the concurrent stabilization of heterogeneous compounds. To overcome these limitations, microencapsulation technology offers an advanced approach by constructing environmentally responsive composite wall systems to entrap core materials, thereby minimizing environmental degradation and facilitating targeted delivery [7].

Sodium alginate (SA) and chitosan (CS) are ideal candidates for constructing such delivery systems. Through intermolecular electrostatic interactions between the anionic carboxyl groups of SA and the cationic amino groups of CS, they form robust polyelectrolyte complex hydrogels widely utilized for protective and controlled-release applications [8].

Several studies have indicated that delivery systems based on biopolymers hold significant application potential for bioactive compounds derived from jujube trees. For example, research has demonstrated the successful microencapsulation of ZJPA, thereby enhancing its stability during in vitro digestion [7]. Additionally, the CS-SA system, as a classical polyelectrolyte complex, is widely employed in studies on the encapsulation of various bioactive compounds due to its excellent biocompatibility, pH-responsive properties, and ability to form robust hydrogel networks under mild conditions [8]. Specifically, CS-SA hydrogels show great promise for protecting sensitive core materials from the harsh acidic environment of the stomach and enabling their controlled release in the intestine. However, despite these advances, most current research still primarily focuses on the encapsulation of single-component systems. The co-encapsulation of multiple bioactive compounds—particularly the combination of macromolecules (e.g., polysaccharides) and small molecules (e.g., cyclic nucleotides)—remains relatively underexplored [9]. Furthermore, there is limited research on the potential enhanced effects between different classes of bioactive compounds and their overall stability within a single delivery matrix.

Therefore, this study aimed to fabricate an advanced chitosan–sodium-alginate (CS-SA) composite hydrogel bead system to co-encapsulate ZJPA and cAMP. The preparation parameters were systematically optimized using single-factor experiments combined with response surface methodology (RSM). Furthermore, the structural characteristics, physicochemical properties, enhanced antioxidant activity, and in vitro gastrointestinal release behavior of the co-encapsulated hydrogel beads were thoroughly evaluated. This study seeks to provide a high-efficiency, environmentally responsive delivery platform for stabilizing and intelligently releasing jujube-derived bioactive compounds, thereby expanding their application potential in functional food development.

## 2. Results and Discussion

### 2.1. Resuls and Analysis of Single-Factor Experiments

#### 2.1.1. Effect of CaCl_2_ Concentration (%) on the Microencapsulation Yield of ZJPA-cAMP/CS-SA Hydrogel Beads

As shown in Figure 1a, the microencapsulation yield (MEY) of the hydrogel beads initially increased and subsequently decreased with increasing CaCl_2_ concentration. The maximum MEY of 93.34% was achieved at a CaCl_2_ concentration of 3%. This improvement was mainly attributed to the ionic crosslinking between Ca^2+^ and sodium alginate, where an appropriate Ca^2+^ concentration promoted the formation of calcium alginate networks, thereby enhancing hydrogel structural stability and improving cAMP retention within the gel matrix [10].

However, further increasing the CaCl_2_ concentration beyond 3% may result in saturation of available alginate binding sites and excessive surface crosslinking, restricting the diffusion of core materials into the hydrogel network and consequently reducing MEY. Therefore, 3% CaCl_2_ was selected as the optimal crosslinking condition.

#### 2.1.2. Effect of CS Concentration (%) on the Microencapsulation Yield of ZJPA-cAMP/CS-SA Hydrogel Beads

As shown in Figure 1b, the MEY of the hydrogel beads initially increased and subsequently decreased with increasing CS concentration. The highest MEY (92.74%) was achieved at a CS concentration of 0.5% (*w*/*v*). CS mainly influenced the formation of the outer coating layer of hydrogel beads, and an appropriate CS concentration contributed to the construction of an effective coating structure, thereby improving MEY [11]. However, further increasing the CS concentration may result in an excessively thick coating layer, reducing the loading capacity of the hydrogel beads, whereas insufficient CS concentration may fail to provide adequate encapsulation. Therefore, 0.5% (*w*/*v*) CS was selected as the optimal concentration for hydrogel bead preparation.

#### 2.1.3. Effect of SA Concentration (%) on the Microencapsulation Yield of ZJPA-cAMP/CS-SA Hydrogel Beads

As shown in Figure 1c, SA concentration significantly affected the MEY of the hydrogel beads. The MEY initially increased and subsequently decreased with increasing SA concentration, reaching a maximum value of 93.35% at 3% (*w*/*v*) SA. When the SA concentration was below 3%, increasing SA concentration promoted the ion exchange and crosslinking between Ca^2+^ and sodium alginate, facilitating the formation of water-insoluble calcium alginate networks and improving hydrogel stability, thereby enhancing MEY. However, further increasing the SA concentration beyond 3% significantly increased solution viscosity due to the strong gel-forming ability of sodium alginate [12]. The highly viscous system tended to adhere to the syringe and container during extrusion, which hindered droplet formation and consequently reduced MEY. Therefore, 3% (*w*/*v*) SA was selected as the optimal concentration.

#### 2.1.4. Effect of the Core-to-Wall Ratio (g:g) on the Microencapsulation Yield of ZJPA-cAMP/CS-SA Hydrogel Beads

As shown in Figure 1d, the core-to-wall ratio significantly affected the MEY of the hydrogel beads. When the core-to-wall ratio was higher than 1:6, the MEY remained relatively low due to the insufficient amount of wall materials required for complete encapsulation of the core compounds. With decreasing core-to-wall ratio, the MEY gradually increased and reached a maximum value of 95.95% at a ratio of 1:6. However, further reduction in the core-to-wall ratio (<1:6) resulted in a decline in MEY, which may be attributed to the excessive amount of wall materials increasing the system viscosity and affecting droplet formation during gelation, thereby reducing microencapsulation yield [13]. Therefore, a core-to-wall ratio of 1:6 was selected as the optimal condition.

### 2.2. Results and Analysis of Response Surface Methodology

#### 2.2.1. Experimental Design and Results of the Response Surface Methodology

Based on the results of the single-factor experiments, a Box–Behnken design (BBD) was employed to optimize the preparation conditions of the ZJPA-cAMP/CS-SA hydrogel beads using MEY as the response value (Y). Response surface methodology (RSM) was applied to determine the optimal preparation conditions for achieving maximum MEY. The experimental design and corresponding results are presented in Table 1. Multiple regression analysis was performed on the experimental data using Design-Expert 13 software, and the following second-order polynomial regression equation was obtained:

Y = 97.30 + 0.2875A − 0.4308B − 0.3658C + 0.5058D − 0.6900AB + 0.2225AC − 0.1850AD + 0.3750BC − 0.2275BD + 0.5800CD − 1.69A_2_ − 1.81B2 − 1.03C_2_ − 1.68D_2_.

The adequacy of the regression model was evaluated by analysis of variance (ANOVA), and the results are presented in Table 2. The model was highly significant, as indicated by a large F-value of 20.29 and a very low probability value (*p* < 0.0001). This confirms that the terms in the model have a significant effect on the response. The coefficient of determination (R^2^) is 0.9530, indicating that the model accounts for 95.30% of the variability in microencapsulation yield. The adjusted R^2^ value (0.9060) is in good agreement with the predicted R^2^ value, confirming a strong correlation between the experimental and predicted values. The close alignment of the adjusted R^2^ value suggests that the model does not suffer from overfitting. The F-statistic for model fit is not significant (*p* = 0.3777 > 0.05). This indicator serves as a key criterion for assessing whether the model fit is satisfactory, as it suggests that the deviation between the model and the experimental data stems from random noise rather than from any systematic inadequacy in the model’s ability to capture the true underlying relationship. Overall, these statistical analyses demonstrate that the established second-order polynomial model is both sufficient and reliable for predicting the microencapsulation yield of ZJPA-cAMP/CS-SA hydrogel beads within the experimental range.

In the regression model, the linear terms B and D showed extremely significant effects on MEY (*p* < 0.01), and all quadratic terms (A^2^, B^2^, C^2^, and D^2^) were highly significant (*p* < 0.01). The remaining terms showed no significant effects on MEY (*p* > 0.05). According to the F-values, the relative influence of the investigated factors on the MEY of ZJPA-cAMP/CS-SA hydrogel beads followed the order of core-to-wall ratio (D) > CS concentration (B) > SA concentration (C) > CaCl_2_ concentration (A). Among these factors, CS concentration (B) and core-to-wall ratio (D) exhibited extremely significant effects on MEY.

Furthermore, based on the results in Table 2 and Figure 2, significant interaction effects were observed between CaCl_2_ concentration and CS concentration, as well as between SA concentration and core-to-wall ratio. In particular, the interaction between CaCl_2_ concentration and CS concentration showed an extremely significant effect on the MEY of the hydrogel beads (*p* < 0.01). However, the interaction terms of CaCl_2_ concentration × SA concentration, CaCl_2_ concentration × core-to-wall ratio, CS concentration × SA concentration, and CS concentration × core-to-wall ratio showed no significant effects on MEY (*p* > 0.05). The significant interaction between CaCl_2_ and CS concentrations suggests an enhanced balance between internal ionic crosslinking and external surface coating. At low concentrations of both factors, the insufficient crosslinking density and thin polyelectrolyte complex layer result in a loose structure and low MEY. Conversely, at high concentrations, excessive crosslinking and a thick CS coating may hinder the diffusion of core materials, reducing the loading capacity. The maximum MEY is achieved when a stable internal network (provided by optimal CaCl_2_) is combined with an effective surface sealing layer (provided by optimal CS).

The significant interaction between CaCl_2_ and CS concentrations suggests an enhanced balance between internal ionic crosslinking and external surface coating. At low concentrations of both factors, the insufficient crosslinking density and thin polyelectrolyte complex layer result in a loose structure and low MEY. Conversely, at high concentrations, excessive crosslinking and a thick CS coating may hinder the diffusion of core materials, reducing the loading capacity. The maximum MEY is achieved when a stable internal network (provided by optimal CaCl_2_) is combined with an effective surface sealing layer (provided by optimal CS).

#### 2.2.2. Interaction Effects of Factors on Microencapsulation Yield

The response surface plots and contour plots for the interactions among the investigated factors are shown in Figure 2. These plots were used to evaluate the effects of the interactions between two variables on the MEY of ZJPA-cAMP/CS-SA hydrogel beads. A steeper response surface slope and denser contour lines indicate a greater influence of the corresponding factors on MEY. In addition, an elliptical contour plot suggests the presence of an interaction effect between two variables, whereas a contour plot approaching a circular shape indicates a weaker interaction between the factors.

#### 2.2.3. Determination of Optimal Preparation Conditions for Maximum Microencapsulation Yield

The optimal preparation conditions for ZJPA-cAMP/CS-SA hydrogel beads were predicted using the quadratic regression model established by response surface methodology. The predicted optimal conditions were as follows: CaCl_2_ concentration of 3.10%, CS concentration of 0.47%, SA concentration of 2.84%, and a core-to-wall ratio of 1:5.7. Under these conditions, the predicted MEY of the hydrogel beads reached 97.41%. To verify the reliability of the predicted model, validation experiments were performed under practical preparation conditions. The optimized parameters were adjusted to CaCl_2_ concentration of 3%, CS concentration of 0.5%, SA concentration of 3%, and a core-to-wall ratio of 1:6. Three independent replicate experiments were conducted under these conditions, and the actual cAMP- MEY was determined to be 97.79% ± 0.20%, which was in good agreement with the predicted value. These results demonstrated the validity of the response surface model and confirmed that the optimized preparation conditions were reliable and applicable for the fabrication of ZJPA-cAMP/CS-SA hydrogel beads.

### 2.3. Physicochemical Properties of ZJPA-cAMP/CS-SA Hydrogel Beads

#### 2.3.1. Main Physicochemical Properties

Table 3 shows that the water-holding capacity of the ZJPA-cAMP/CS-SA hydrogel beads is 72.93% ± 3.93%, indicating that the CS-SA network possesses strong hydrophilicity and water-retention capability. The angle of repose is 33.02° ± 1.99°, which is less than 45°, indicating good flowability of the lyophilized powder; the bulk density is 0.60 ± 0.02 g/cm^3^, representing a moderate value that is favorable for filling, packaging, and processing. The solubility is 38.16% ± 0.75%, which is relatively low, suggesting that the crosslinked network is stable and exhibits good water resistance—properties that help prevent premature leakage of the core material and support gastric protection as well as intestinal controlled release. Overall, these hydrogel beads combine excellent water-holding capacity, good powder flowability, and structural stability, making them suitable as delivery carriers for ZJPA and cAMP in functional food applications.

#### 2.3.2. Macroscopic Appearance and Color Characteristics of ZJPA-cAMP/CS-SA Hydrogel Beads

Figure 3 illustrates the surface morphology changes in blank hydrogel beads and ZJPA-cAMP/CS-SA hydrogel beads under different drying conditions. In the fresh state, the blank hydrogel beads (A_1_ and a_1_) exhibited a white color, smooth surface, and regular spherical morphology. The ZJPA-cAMP/CS-SA hydrogel beads (B_1_ and b_1_) also maintained a well-defined spherical structure, with L*, a*, and b* values of 34.17, 4.40, and 26.19, respectively, showing a distinct yellow appearance mainly attributed to the intrinsic color of jujube polysaccharides.

After oven-drying at 30 °C, both blank and ZJPA-cAMP/CS-SA hydrogel beads exhibited noticeable shrinkage, reduced volume, and surface wrinkling. For the ZJPA-cAMP/CS-SA hydrogel beads, the L* value decreased to 25.77, while the a* value increased to 12.66 and the b* value decreased to 2.50. The ΔE* value reached 26.45, indicating considerable color variation after oven-drying.

In contrast, vacuum freeze-dried hydrogel beads showed less shrinkage, better preservation of spherical morphology, and the formation of a porous surface structure. The ZJPA-cAMP/CS-SA hydrogel beads after freeze-drying exhibited L*, a*, and b* values of 28.64, 5.56, and 15.55, respectively, with a lower ΔE* value of 12.05, indicating better color retention compared with oven-drying. Table 4 demonstrated that vacuum freeze-drying was more effective in maintaining the morphological integrity and color characteristics of hydrogel beads, which may contribute to improved product quality stability [14].

#### 2.3.3. Diameter Analysis of Hydrogel Beads

As shown in Figure 4a, the diameter variations in blank hydrogel beads and ZJPA-cAMP/CS-SA hydrogel beads under different drying conditions were investigated. For both types of hydrogel beads, the diameters in the fresh state were significantly larger than those after drying, while the oven-dried samples exhibited the smallest diameters. These results indicated that water loss during drying induced shrinkage of the hydrogel network. Compared with oven-drying, vacuum freeze-drying was more effective in preserving the structural integrity of hydrogel beads by reducing network collapse and deformation [15].

Under all three conditions, the diameters of ZJPA-cAMP/CS-SA hydrogel beads were consistently larger than those of blank hydrogel beads, indicating that the incorporation of ZJPA and cAMP did not negatively affect bead formation. Instead, the interactions between the encapsulated compounds and the CS-SA matrix may contribute to the stabilization of the hydrogel network, resulting in larger bead diameters. These findings suggested good compatibility between the core materials and wall matrix, which supported the structural stability of the composite hydrogel beads.

#### 2.3.4. Rheological Properties of Hydrogel Beads

As shown in Figure 4b, the storage modulus (G′) of both blank hydrogel beads and ZJPA-cAMP/CS-SA hydrogel beads increased with increasing angular frequency. The G′ values remained higher than the loss modulus (G″), indicating that both hydrogel systems exhibited typical elastic gel-like characteristics dominated by solid behavior [16]. The blank hydrogel beads showed consistently higher G′ values than the ZJPA-cAMP/CS-SA hydrogel beads, suggesting stronger network elasticity and structural strength. The incorporation of ZJPA and cAMP may alter the structural organization of the CS-SA matrix, reducing the elastic response and resulting in a softer gel behavior of the composite hydrogel beads [17].

#### 2.3.5. Textural Properties of Hydrogel Beads

As shown in Table 5, the hardness, cohesiveness, springiness, gumminess, and chewiness of ZJPA-cAMP/CS-SA hydrogel beads were lower than those of blank hydrogel beads. Among these parameters, the decreases in hardness, springiness, and chewiness were significant. This phenomenon may be attributed to the incorporation of ZJPA and cAMP, which modified the structural characteristics of the CS-SA gel network. The interactions between the encapsulated compounds and the wall matrix reduced the rigidity and elasticity of the gel system, resulting in softer textural properties of the composite hydrogel beads. Such soft texture characteristics are more suitable for the processing and consumption requirements of food-grade encapsulation systems [18].

#### 2.3.6. Moisture Content Analysis of Hydrogel Beads

As shown in Figure 4c, both blank hydrogel beads and ZJPA-cAMP/CS-SA hydrogel beads exhibited high moisture contents (>90%) in the fresh state, with no significant differences between the two groups. After drying treatment, the moisture contents of both hydrogel bead systems decreased significantly, reaching approximately 15% after oven-drying and below 5% after vacuum freeze-drying. These results indicated that drying treatments effectively removed water from the hydrogel beads. Among the investigated methods, vacuum freeze-drying exhibited a stronger dehydration effect, which may contribute to reducing water activity and improving storage stability [19]. Although oven-drying also markedly decreased moisture content, its dehydration effect was relatively lower.

Furthermore, no significant differences in moisture content were observed between blank hydrogel beads and ZJPA-cAMP/CS-SA hydrogel beads under the same drying conditions, indicating that the incorporation of core materials did not significantly affect the water retention or dehydration behavior of the hydrogel beads. Instead, moisture variation was mainly determined by the drying process.

#### 2.3.7. Swelling Degree (SD) Analysis of Hydrogel Beads

As shown in Figure 5a,b, the swelling degree of hydrogel beads under simulated gastric fluid (SGF, pH 3.0) and simulated intestinal fluid (SIF, pH 7.0) conditions gradually increased with prolonged immersion time and reached equilibrium at approximately 4 h. The fresh hydrogel beads exhibited relatively limited swelling throughout the immersion period, which may be attributed to their initially hydrated polymer networks with limited available space for further water uptake [20].

In contrast, both drying treatments significantly enhanced the swelling capacity of hydrogel beads. Among the tested samples, vacuum freeze-dried hydrogel beads exhibited the highest swelling degree throughout the swelling process, reaching 1921% in SGF and over 2000% in SIF, followed by oven-dried samples. The superior swelling performance of freeze-dried beads was mainly attributed to the porous structure generated by ice crystal sublimation, which increased the exposure of hydrophilic functional groups and provided more water-binding sites. In comparison, oven-drying induced network shrinkage and pore collapse due to capillary forces during water evaporation, thereby reducing water accessibility to hydrophilic groups and resulting in lower swelling capacity [21].

Furthermore, pH conditions markedly influenced the swelling behavior of hydrogel beads. The equilibrium swelling degree under neutral SIF conditions (pH 7.0) was significantly higher than that under acidic SGF conditions (pH 3.0). Under acidic conditions, protonation of amino groups in chitosan and carboxyl groups in alginate reduced the charge density of the polymer network and strengthened intermolecular interactions, thereby restricting network expansion and water penetration. Conversely, under neutral conditions, deprotonation of alginate carboxyl groups generated stronger electrostatic repulsion among polymer chains, promoting network expansion and facilitating water uptake. Consequently, the hydrogel beads exhibited enhanced swelling capacity under intestinal conditions.

#### 2.3.8. DPPH Radical Scavenging Activity Analysis

As shown in Figure 5d, the DPPH radical scavenging activities of ZJPA, cAMP, and ZJPA-cAMP/CS-SA hydrogel beads gradually increased with increasing sample concentration, exhibiting a clear concentration-dependent antioxidant response. In contrast, the DPPH scavenging activity of free cAMP remained relatively stable across the tested concentration range, showing no obvious concentration dependence. This may be attributed to the limited intrinsic radical scavenging capacity of cAMP itself, whereas the enhanced and concentration-dependent activity observed for ZJPA and the hydrogel beads is likely due to the enhanced contribution of polyphenols and other bioactive compounds present in ZJPA.

Further analysis revealed that the antioxidant activity of the composite system increased with increasing ZJPA proportion, suggesting that ZJPA played an important role in maintaining and enhancing the antioxidant performance of the combined system. Among all tested samples, the ZJPA-cAMP/CS-SA hydrogel beads exhibited the highest DPPH radical scavenging activity at a concentration of 1 mg/mL, indicating that the CS-SA hydrogel matrix effectively preserved the antioxidant functionality of the encapsulated bioactive compounds.

Previous studies have demonstrated that jujube polysaccharides contain abundant hydroxyl, carboxyl, and other active groups, which contribute to free radical scavenging through hydrogen donation. Moreover, interactions between polysaccharides and bioactive small molecules may further influence the antioxidant performance of composite systems. These findings are consistent with the results reported by Guo et al. [22], further highlighting the potential of the ZJPA-cAMP co-delivery strategy for enhancing functional activity.

#### 2.3.9. Thermogravimetric Analysis (TGA)

As shown in Figure 5c,d, thermogravimetric analysis (TGA) and derivative thermogravimetric (DTG) curves were obtained to evaluate the thermal stability of ZJPA, cAMP, ZJPA-cAMP, CS, SA, blank hydrogel beads, and ZJPA-cAMP-loaded hydrogel beads. The tested samples exhibited distinct thermal degradation behaviors. Among them, cAMP showed relatively higher thermal resistance, with greater residual mass retention at elevated temperatures. In comparison, ZJPA, CS, and SA exhibited lower degradation temperatures and relatively weaker thermal stability.

Compared with the individual core and wall materials, both blank hydrogel beads and ZJPA-cAMP-loaded hydrogel beads displayed shifts in degradation peak temperatures and weight-loss regions, indicating that encapsulation modified the thermal degradation behavior of the CS-SA system. Notably, the maximum degradation temperature of ZJPA-cAMP-loaded hydrogel beads was higher than that of blank hydrogel beads, suggesting improved thermal stability after incorporation of the core materials. This enhancement may be attributed to the interactions between ZJPA-cAMP and the CS-SA matrix, including hydrogen bonding and ionic interactions, which contributed to the formation of a more stable composite network [23]. Moreover, the CS-SA wall matrix could act as a protective barrier against thermal stress, thereby improving the thermal tolerance of encapsulated bioactive compounds. These results demonstrated that the CS-SA hydrogel system effectively enhanced the thermal stability of ZJPA-cAMP, supporting its potential application in functional food processing.

### 2.4. Structural Characterization of ZJPA-cAMP/CS-SA Hydrogel Beads

#### 2.4.1. X-Ray Diffraction (XRD) Analysis

As shown in Figure 6a,b, the SA, CS, blank hydrogel beads, and hydrogel beads loaded with ZJPA–cAMP all exhibit broad diffuse diffraction peaks without any distinct sharp crystalline reflection peaks; this indicates that these components predominantly exist in an amorphous state. After ion crosslinking and incorporation of ZJPA–cAMP, the characteristic peaks of SA and CS exhibit slight shifts in position and reduced intensity; this phenomenon may be attributed to ion interactions and intermolecular hydrogen bonding effects that have altered the arrangement of the polymer chains. Furthermore, the X-ray diffraction (XRD) pattern of the ZJPA–cAMP-loaded hydrogel beads closely resembles that of the blank hydrogel beads, and no characteristic crystalline peaks for cAMP or ZJPA were detected. This suggests that the active compounds are likely dispersed into the CS-SA network in an amorphous or molecularly dissolved form, rather than existing as independent crystalline domains, although the possibility of their crystalline content being below the XRD detection limit cannot be entirely excluded. The incorporation of ZJPA-cAMP did not disrupt the amorphous structure of the hydrogel matrix; this finding is consistent with the formation of a stable composite delivery system. Additionally, the experimentally determined high microencapsulation yield (MEY) of cAMP further supports the effective entrapment of the core material within this matrix [24].

#### 2.4.2. Fourier Transform Infrared (FTIR) Spectroscopy Analysis

FTIR spectroscopy was performed to investigate the molecular interactions among the ZJPA-cAMP core material, CS-SA wall matrix, and composite hydrogel beads. As shown in Figure 6c, ZJPA-cAMP, SA, CS, blank hydrogel beads, and ZJPA-cAMP-loaded hydrogel beads exhibited their corresponding characteristic absorption bands.

The ZJPA-cAMP complex showed a broad absorption band at 3432 cm^−1^ associated with hydroxyl (-OH) stretching vibrations, while the peak around 1610 cm^−1^ was related to carbonyl-associated vibrations. The absorption bands in the range of 1094–1029 cm^−1^ were mainly attributed to C-O stretching vibrations [25]. SA and CS exhibited their typical absorption peaks corresponding to carboxylate groups, hydroxyl/amino groups, and amide groups, respectively [26].

Compared with the original wall materials, the characteristic peaks of SA and CS in blank hydrogel beads showed slight shifts. Specifically, the hydroxyl/amino-related band shifted from 3420/3397 cm^−1^ to 3400 cm^−1^, and the carboxylate-related peak shifted from 1611 cm^−1^ to 1605 cm^−1^, indicating the formation of intermolecular interactions between CS and SA during hydrogel network construction. The absence of new characteristic peaks suggested that no new covalent bonds were generated during the crosslinking process [27].

The FTIR spectrum of ZJPA-cAMP-loaded hydrogel beads retained the characteristic features of SA, CS, and ZJPA-cAMP. After encapsulation, slight shifts in hydroxyl and carboxyl/amide-related bands were observed, suggesting the presence of hydrogen bonding and electrostatic interactions between ZJPA-cAMP and the CS-SA matrix. Meanwhile, no new covalent bond-related peaks appeared, indicating that ZJPA-cAMP was physically incorporated into the CS-SA hydrogel network through non-covalent interactions. This interpretation is in agreement with the structural characterization data, corroborating the successful incorporation of the core materials within the hydrogel beads via non-covalent mechanisms.

#### 2.4.3. Scanning Electron Microscopy (SEM) Analysis

As shown in Figure 7, the freeze-dried hydrogel beads exhibit an irregular flaky morphology, with distinct wrinkles and depressions on their surfaces. These features primarily arise from the contraction of the hydrogel network structure during water sublimation in the freeze-drying process [28]. The surfaces of the hydrogel beads are relatively dense and continuous, showing no obvious pores or cracks. This intact surface morphology indicates the formation of a dense CS-SA matrix, providing a favorable structural foundation for the physical encapsulation of the core material. The presence of localized wrinkles and stacked structures demonstrates that the freeze-drying process did not cause severe structural damage to the polymer network [29].

Overall, the SEM observations reveal that the prepared hydrogel beads possess a stable network morphology, confirming the structural integrity of the CS-SA system as a potential delivery carrier.

### 2.5. In Vitro Simulated Gastrointestinal Release Behavior

As shown in Figure 6d, the simulated gastrointestinal environment exerted a pronounced regulatory effect on the release behavior of cAMP from ZJPA-cAMP-loaded hydrogel beads. During the oral digestion stage, both the unencapsulated ZJPA-cAMP complex solution and the ZJPA-cAMP/CS-SA hydrogel beads exhibited minimal cAMP release, indicating that the hydrogel bead system effectively maintained the stability of the encapsulated bioactive compounds during the initial digestion process.

Upon exposure to simulated gastric fluid (SGF, 2–120 min), cAMP in the unencapsulated complex solution was rapidly released, whereas the release of cAMP from the hydrogel beads was significantly delayed, demonstrating an effective gastric protective effect. This behavior was mainly attributed to the contraction of the CS-SA polyelectrolyte network and enhanced intermolecular interactions under acidic conditions, which generated a compact barrier structure and reduced direct exposure of cAMP to the gastric environment [30].

After transferring to simulated intestinal fluid (SIF, 120–240 min), the increased pH promoted deprotonation of polymer chains and enhanced electrostatic repulsion among negatively charged alginate groups, resulting in hydrogel network expansion and improved water penetration. Consequently, cAMP encapsulated within the hydrogel beads was gradually released from the matrix, exhibiting a distinct pH-responsive release behavior. This result was consistent with the swelling behavior observed under neutral conditions.

In contrast, the unencapsulated ZJPA-cAMP complex solution reached release equilibrium after approximately 150 min, indicating its limited protective capacity against gastrointestinal environmental changes. Overall, the ZJPA-cAMP/CS-SA hydrogel beads effectively delayed cAMP release under gastric conditions while promoting sustained release under intestinal conditions, demonstrating excellent pH-responsive gastrointestinal delivery performance and providing a promising strategy for the application of jujube-derived bioactive compounds in functional foods [31].

## 3. Conclusions and Prospects

In this study, a pH-responsive CS-SA composite hydrogel bead system was successfully developed as a platform for the co-delivery of jujube polysaccharides (ZJPA) and cyclic adenosine monophosphate (cAMP). With respect to quantitative encapsulation, the optimized hydrogel beads exhibited a high microencapsulation yield (MEY) of 97.79% for cAMP, as independently determined by UV spectrophotometry, demonstrating the excellent loading capacity of the CS-SA matrix for cAMP.

Regarding structural evidence, structural characterization revealed that ZJPA and cAMP were successfully incorporated into the CS-SA network through electrostatic interactions and hydrogen bonding without the formation of new covalent bonds, resulting in a stable amorphous encapsulation structure. Furthermore, the hydrogel beads exhibited distinct pH-responsive swelling and gastrointestinal release behaviors. Quantitatively, only 17.20% of cAMP was released in the simulated gastric phase (2–120 min), confirming the protective effect of the compact CS-SA network against premature release, whereas sustained release of cAMP was observed under intestinal conditions, reaching 58.31% at 240 min. Importantly, the co-encapsulation of ZJPA and cAMP maintained their enhanced antioxidant activity, suggesting that the co-delivery strategy is a promising approach for preserving the functional properties of these bioactive compounds.

Overall, this study provides a multifunctional hydrogel bead platform that integrates the potential for bioactive synergy, structural protection, and pH-responsive gastrointestinal delivery, offering a promising strategy for enhancing the stability, functionality, and application potential of jujube-derived bioactive compounds in functional foods.

Although the developed hydrogel beads demonstrated excellent in vitro performance, further investigations are required to promote their practical application. Future studies should focus on evaluating long-term storage stability, bioaccessibility, and in vivo functional efficacy through cellular and animal models. In particular, since ZJPA was not independently quantified in the present study, the encapsulation and release of ZJPA could not be evaluated quantitatively, and the development of a validated quantitative method for ZJPA in the beads will be a priority in future work. In addition, optimization of biopolymeric formulations, scale-up production, and processing compatibility should be further explored to facilitate the translation of this delivery system from laboratory research to commercial functional food applications.

## 4. Materials and Methods

### 4.1. Materials and Instruments

The jujube fruits used in this study were purchased from the 11th Regiment of Alar City, Xinjiang, China. Jujube polysaccharides (ZJPA) were extracted from jujube fruits using hot water extraction followed by ethanol precipitation. Standard-grade cyclic adenosine monophosphate (cAMP) and trypsin (1:3000) (Solarbio Science & Technology Co., Ltd., Beijing, China); analytical-grade sodium alginate (SA) (Aladdin Biochemical Technology Co., Ltd., Shanghai, China); chitosan (CS) (Yuanye Bio-Technology Co., Ltd., Shanghai, China); calcium chloride (Aopusheng Chemical Co., Ltd., Tianjin, China); absolute ethanol (Zhiyuan Chemical Reagent Co., Ltd., Tianjin, China); glacial acetic acid (Xinbote Chemical Co., Ltd., Tianjin, China); pepsin (1:4000) (Ruige Biotechnology Co., Ltd., Shanghai, China);spectroscopic-grade potassium bromide (KBr) (Boten Shengda Technology Development Co., Ltd., Tianjin, China).

UV-Vis spectrophotometer (Model 725, Shanghai Lengguang Technology Co., Ltd., Shanghai, China); modular advanced rheometer (MC2102e, Anton Paar GmbH, Graz, Austria); texture analyzer (TMS-PILOT, Sheng Heng Tai Technology Co., Ltd., Beijing, China); mid-infrared spectrometer (Frontier, Bruker Optics, Ettlingen, Germany); fixed-speed vortex mixer (MX-F, DLAB Scientific Co., Ltd., Beijing, China); thermostatic magnetic heating stirrer (DF-101S, Wuhan Delixiang Instrument Equipment Co., Ltd., Wuhan, China); colorimeter (NR60CP, 3nh Technology Co., Ltd., Guangzhou, China); digital caliper (DYX91150, Shanghai Deyixing Tools Group Co., Ltd., Shanghai, China); scanning electron microscope (Gemini SEM 460, Carl Zeiss AG, Oberkochen, Germany); X-ray diffractometer (Ultima IV, Rigaku, Japan); shaking incubator (HZQX300C, Shanghai LAMPADE Instrument Co., Ltd., Shanghai, China).

### 4.2. Experimental Methods

#### 4.2.1. Preparation Procedure for ZJPA-cAMP/CS-SA Hydrogel Beads

The detailed manufacturing process is illustrated in Figure 8, and the specific steps are as follows:

#### 4.2.2. Preparation of the SA Wall Solution

A predetermined amount of SA was dissolved in distilled water in an 80 °C water bath to obtain an SA solution at the desired concentration. The solution was allowed to cool to room temperature before use.

#### 4.2.3. Preparation of the ZJPA-cAMP Core/SA Wall Mixture

According to the predetermined core-to-wall ratio, a mixture of ZJPA and standard-grade cAMP (SolarbioScience & Technology Co., Ltd., Beijing, China) was used as the core material. An appropriate amount of this ZJPA-cAMP mixture was dispersed in distilled water and ultrasonically treated at 40 °C and 200 W for 30 min. The resulting suspension was then mixed with the SA solution and stirred thoroughly to obtain a homogeneous core-wall mixture, which was allowed to stand before further use.

#### 4.2.4. Preparation of the CS-CaCl_2_ Coagulation Bath

CS was dissolved in a 1% (*v*/*v*) glacial acetic acid solution, and then the pH was adjusted to approximately 5.0 using 5% (*w*/*v*) citric acid to obtain the desired concentration. Calcium chloride (CaCl_2_) solution at the predetermined concentration was prepared separately using distilled water. Subsequently, the two solutions were mixed to form the CS-CaCl_2_ coagulation bath.

#### 4.2.5. Preparation of ZJPA-cAMP/CS-SA Hydrogel Beads

Refer to the method proposed by Gao et al. [32] for preparing ZJPA-cAMP/CS-SA hydrogel beads (Figure 8). The CS-CaCl_2_ coagulation bath was maintained at 25 °C under continuous magnetic stirring. The prepared ZJPA-cAMP/SA core-wall mixture was transferred into a 5 mL syringe fitted with a 0.5 mm needle and added dropwise into the coagulation bath. Hydrogel beads were formed immediately through ionotropic gelation and allowed to crosslink for 30 min. After gelation, the coagulation bath was discarded, and the hydrogel beads were thoroughly rinsed with deionized water to remove residual solution from the surface. The beads were then vacuum-filtered to remove excess surface moisture and transferred to Petri dishes for subsequent analyses.

#### 4.2.6. Establishment of the cAMP Calibration Curve

The calibration curve of cAMP was established according to the method reported by Davi et al. [8] with slight modifications. Briefly, 0.0025 g of cAMP standard was accurately weighed, dissolved in deionized water, and transferred to a 100 mL volumetric flask to prepare a standard stock solution with a concentration of 25 µg/mL. The stock solution was subsequently diluted with deionized water to obtain a series of standard solutions with concentrations of 5, 10, 15, 20, and 25 µg/mL. Deionized water was used as the blank control. The absorption spectrum of the cAMP standard solution was scanned using a UV-Vis spectrophotometer, and the maximum absorption wavelength was determined to be 259 nm. The absorbance values of cAMP standard solutions at different concentrations were measured at 259 nm, and the calibration curve was constructed by plotting absorbance against cAMP concentration. The regression equation was obtained as follows: Y = 0.0412X + 0.0236 (R^2^ = 0.99844).

#### 4.2.7. Determination of Microencapsulation Yield (MEY)

The MEY of the hydrogel beads was determined according to the method described by Huang et al. [33] with slight modifications. Briefly, 1 g of hydrogel beads was accurately weighed, cut into small pieces, and mixed with 5 mL of absolute ethanol. The mixture was thoroughly ground and vortexed for 3 min to completely disrupt the hydrogel matrix and release the encapsulated cAMP, followed by centrifugation at 8000 rpm for 10 min. The supernatant was collected, and the absorbance was measured at 259 nm using a UV-Vis spectrophotometer (Model 725, Shanghai Lengguang Technology Co., Ltd., Shanghai, China). The mass of total cAMP recovered was calculated based on the established cAMP calibration curve. The MEY of the hydrogel beads was calculated using the following equation:

(1) $$\mathrm{MEY}\left(\%\right)=\left(\frac{m_{1}}{m_{2}}\right)\times 100$$

where MEY represents the microencapsulation yield (%), m_1_ represents the mass of cAMP recovered from the disrupted hydrogel beads (g), and m_2_ represents theoretical total mass of cAMP added during the preparation process (g).

Limitations: It should be noted that quantitative analysis of cAMP using UV-Vis spectroscopy at 259 nm in composite hydrogel matrices containing CS, SA, and ZJPA inherently possesses certain limitations. Although theoretical spectra indicate that interference from CS and SA at this wavelength is minimal, background absorbance arising from trace matrix degradation products or co-extracted ZJPA components during ethanol lysis cannot be entirely ruled out.

### 4.3. *Single-Factor Experimental Design*

A single-factor experimental design was employed to investigate the effects of different preparation parameters on the MEY of ZJPA-cAMP/CS-SA hydrogel beads while maintaining all other preparation conditions constant. Four independent variables, including CaCl_2_ concentration, CS concentration, SA concentration, and the core-to-wall ratio, were selected for evaluation. According to the single-factor experimental design, each variable was investigated at five predetermined levels. The CaCl_2_ concentrations were set at 1%, 2%, 3%, 4%, and 5%; the CS concentrations were 0.1%, 0.3%, 0.5%, 0.7%, and 0.9%; the SA concentrations were 1%, 2%, 3%, 4%, and 5%; and the core-to-wall ratios were 1:3, 1:4, 1:5, 1:6, and 1:7 (g/g). After each single-factor experiment, the MEY of the hydrogel beads was determined to evaluate the influence of each preparation parameter on the encapsulation performance of ZJPA-cAMP.

The investigated factor ranges and the corresponding five levels were established based on preliminary exploratory experiments. Five experimental levels were selected to achieve an appropriate balance between experimental efficiency and the ability to identify trends in the response variable. The parameter ranges were further refined by excluding conditions that exhibited poor encapsulation performance during the preliminary experiments, thereby ensuring that the optimal preparation conditions were encompassed within the selected experimental region. For each experimental level, three independent batches of hydrogel beads were prepared (*n* = 3), rather than performing analytical replicates on a single batch, to evaluate the overall experimental variability and reproducibility of the hydrogel bead fabrication process.

### 4.4. Response Surface Methodology (RSM) Optimization

Based on the results of single-factor experiments, the response surface methodology (RSM) was employed to optimize the preparation conditions for ZJPA-cAMP/CS-SA hydrogel beads. CaCl_2_ concentration (A), CS concentration (B), SA concentration (C), and the core-to-wall ratio (D) were selected as the independent variables, while the remaining preparation parameters were kept constant. The MEY of the hydrogel beads was used as the response variable. A four-factor, three-level Box–Behnken design (BBD) was adopted, and the complete experimental design matrix together with the coded factor levels is presented in Table 6. A second-order polynomial regression model was established to evaluate the individual and interactive effects of the selected variables on the MEY of the hydrogel beads.

### 4.5. Validation of the RSM Model

Using a second-order polynomial regression model developed by response surface methodology (RSM), the optimal preparation conditions for ZJPA-cAMP/CS-SA hydrogel beads were predicted. The predicted optimal conditions were as follows: calcium chloride concentration of 3.10%, CS concentration of 0.47%, SA concentration of 2.84%, and a core–shell ratio of 1:5.7; under these conditions, the MEY of the predicted hydrogel beads reached 97.41%. Considering the feasibility of actual preparation, the optimal conditions were adjusted to a calcium chloride concentration of 3%, a CS concentration of 0.5%, an SA concentration of 3%, and a core–shell ratio of 1:6. Subsequently, three independent validation experiments were conducted under these optimized conditions to assess the reliability and accuracy of the RSM model.

### 4.6. Determination of Physicochemical Properties of ZJPA-cAMP/CS-SA Hydrogel Beads

#### 4.6.1. Determination of Water-Holding Capacity (WHC)

The Water-Holding Capacity (WHC) of ZJPA-cAMP/CS-SA hydrogel beads was determined using the method described by Lei et al. [34], with minor modifications. The procedure is as follows: a specific amount of fresh hydrogel microsphere sample was placed into a centrifugal ultrafiltration tube and centrifuged at 8000 rpm for 20 min; thereafter, the WHC was calculated using the following formula based on the mass difference between the sample before and after centrifugation.

(2) $$WHC(\%)=(M_{0}-M_{1})/M_{0}\times 100$$

where M_0_ represents the weight of the hydrogel beads and centrifuge tubes before centrifugation (g) and M_1_ represents the weight of the hydrogel beads and centrifuge tubes after centrifugation (g).

#### 4.6.2. Determination of the Angle of Repose

The angle of repose is an important indicator for evaluating the flowability of powder materials. Generally, an angle of repose greater than 45° indicates poor powder flowability. The angle of repose was determined according to the method reported by Guo et al. [35] with slight modifications. The freeze-dried ZJPA-cAMP/CS-SA hydrogel beads were ground into fine powder, and the powder height and radius were measured using the fixed-height funnel method. The angle of repose was calculated according to the following equation:

(3) $$\theta =\mathrm{arc}\;\tan\;\frac{H}{R}$$

where θ represents the angle of repose (°), H represents the height of the powder pile (cm), and R represents the radius of the powder pile (cm).

#### 4.6.3. Determination of Bulk Density

The bulk density of the freeze-dried ZJPA-cAMP/CS-SA hydrogel bead powder was determined according to the method described by Feng et al. [36] with slight modifications. Briefly, a known amount of the freeze-dried hydrogel bead powder was transferred into a graduated cylinder and gently tapped repeatedly until the sample volume remained constant. The final volume was recorded, and the bulk density was calculated as the ratio of the sample mass to its occupied volume.

#### 4.6.4. Determination of Solubility

The solubility of the ZJPA-cAMP/CS-SA hydrogel beads was determined according to the method reported by Zeng et al. [37]. The solubility was calculated using Equation (4).

(4) $$\mathrm{Solubility}\;\left(\%\right)=\left(1-\frac{m_{2}-m_{1}}{(1-w)m}\right)\times 100$$

where m_1_ is the mass of the pre-weighed empty evaporating dish for hydrogel bead insoluble residues, m_2_ is the total mass (g) of the evaporating dish with hydrogel bead insoluble residues dried to constant weight and w is the moisture content of the hydrogel beads (%).

#### 4.6.5. Observation of Morphology and Diameter

The macroscopic morphology and diameter of the ZJPA-cAMP/CS-SA hydrogel beads were evaluated according to the method reported by Li et al. [38] with slight modifications. Fresh hydrogel beads, oven-dried hydrogel beads (30 °C), and freeze-dried hydrogel beads were separately placed in clean Petri dishes and photographed under standardized lighting conditions to observe their macroscopic morphology. The diameters of the fresh, oven-dried (30 °C), and freeze-dried hydrogel beads were measured using a digital caliper.

#### 4.6.6. Determination of Color Parameters

The color parameters of fresh, oven-dried (30 °C), and freeze-dried ZJPA-cAMP/CS-SA hydrogel beads were determined using a colorimeter according to the method reported by Han et al. [39] with slight modifications. The CIELab color system was used to characterize the color properties of the hydrogel beads. The L* value represents lightness, ranging from 0 (black) to 100 (white). The a* value indicates the position on the red-green axis, with positive and negative values representing reddish and greenish colors, respectively. The b* value represents the position on the yellow-blue axis, with positive and negative values corresponding to yellowish and bluish colors, respectively. The total color difference (ΔE*) was calculated according to Equation (5).

(5) $${\Delta E}^{*}=\sqrt{{\left(\mathrm{Ln}-L0\right)}^{2}+{\left(\mathrm{an}-a0\right)}^{2}+{\left(\mathrm{bn}-b0\right)}^{2}}$$

where Ln*, an*, and bn* represent the L*, a*, and b* values of the dried hydrogel beads after different drying treatments, respectively, and L0*, a0*, and b0* represent the corresponding values of the fresh hydrogel beads.

#### 4.6.7. Rheological Property Analysis

The rheological properties of the ZJPA-cAMP/CS-SA hydrogel beads were determined using an Anton Paar rheometer according to the method described by Li et al. [40] with slight modifications. Steady shear measurements were performed at 25 °C using a 50 mm parallel-plate geometry (PP50). The apparent viscosity of the samples was recorded over a shear rate range of 0.1–10 s^−1^. Dynamic frequency sweep measurements were subsequently conducted at 25 °C over an angular frequency range of 0.1–10 rad/s to determine the storage modulus (G′) and loss modulus (G″) of the fresh ZJPA-cAMP/CS-SA hydrogel beads.

#### 4.6.8. Texture Profile Analysis

The texture profile of fresh ZJPA-cAMP/CS-SA hydrogel beads was determined according to the method described by Hu et al. [41] with slight modifications using a texture analyzer. The texture parameters, including hardness, cohesiveness, springiness, gumminess, and chewiness, were evaluated. The test conditions were set as follows: probe speed, 60 mm/min; load cell capacity, 25 N; compression ratio, 50%; return distance, 10 mm; and a P/5 cylindrical probe was used. For each measurement, 40 fresh hydrogel beads were carefully selected and uniformly arranged in close contact at the center of the sample platform. Each sample was analyzed in triplicate, and the average value was reported.

#### 4.6.9. Determination of Moisture Content

The moisture content of the ZJPA-cAMP/CS-SA hydrogel beads was determined according to the method reported by Najjaa et al. [42] with slight modifications. Briefly, a certain amount of fresh hydrogel beads was accurately weighed and dried in an oven at 105 °C until a constant weight was reached. The moisture content was calculated based on the percentage of weight loss before and after drying.

#### 4.6.10. Determination of Swelling Degree (SD)

The swelling behavior of fresh, oven-dried (30 °C), and freeze-dried ZJPA-cAMP/CS-SA hydrogel beads was evaluated according to the method reported by [43] with slight modifications. The hydrogel beads were immersed in phosphate-buffered saline (PBS) solutions at pH 3.0 and 7.0 and maintained at 37 °C. At predetermined time intervals, the swollen hydrogel beads were removed, gently blotted to eliminate excess surface moisture, and accurately weighed. The swelling degree (SD) was calculated using the following Equation (6):

(6) $$\mathrm{SD}\left(\%\right)=\left(M_{1}-M_{2}\right)/M_{2}\times 100$$

where M_1_ represents the weight of the hydrogel beads after swelling (g) and M_2_ represents the initial weight of the hydrogel beads before swelling (g).

#### 4.6.11. Determination of DPPH Radical Scavenging Activity

The DPPH radical scavenging activity was determined according to the method reported by Huang et al. [44] with slight modifications. Specifically, DPPH (10.0 mg) was accurately weighed and dissolved in anhydrous ethanol to obtain a 0.04 mg/mL (approximately 0.1 mmol/L) DPPH solution, which was freshly prepared and protected from light before use. In our formulation, ZJPA solution and cAMP solution were prepared at final concentrations of 0.2, 0.4, 0.6, 0.8, and 1.0 mg/mL, and ZJPA and cAMP were complexed at a fixed mass ratio of 4:1 (*w*/*w*). The ZJPA–cAMP/CS–SA hydrogel bead suspensions were prepared at total ZJPA-cAMP concentrations of 0.2, 0.4, 0.6, 0.8, and 1.0 mg/mL (based on the total mass of encapsulated ZJPA and cAMP); accordingly, the actual concentrations of each active compound in the loaded bead samples were ZJPA at 0.16, 0.32, 0.48, 0.64, and 0.80 mg/mL, and cAMP at 0.04, 0.08, 0.12, 0.16, and 0.20 mg/mL, respectively. A certain volume of ZJPA solution, cAMP solution, and ZJPA–cAMP/CS–SA hydrogel bead suspension was mixed with an equal volume of DPPH solution (2 mL of each solution) and incubated in the dark at room temperature for 30 min. The absorbance was measured at 517 nm using a UV–Vis spectrophotometer and recorded as A_1_. Ethanol was used instead of the sample solution to determine the absorbance of the DPPH solution (A_2_), while ethanol was used instead of the DPPH solution to determine the absorbance of the sample solution (A_0_). Vitamin C was used as the positive control. The DPPH radical scavenging activity was calculated using Equation (7):

(7) $$\mathrm{DPPH}\;\mathrm{radical}\;\mathrm{scavenging}\;\mathrm{activity}\;\left(\%\right)=1-\left(A_{1}-A_{0}\right)/A_{2}\times 100$$

where A_1_ represents the absorbance of the sample solution mixed with DPPH solution, A_2_ represents the absorbance of the ethanol mixed with DPPH solution, and A_0_ represents the absorbance of the sample solution mixed with ethanol.

#### 4.6.12. Thermogravimetric Analysis (TGA)

The thermal stability of the ZJPA-cAMP/CS-SA hydrogel beads was evaluated using thermogravimetric analysis (TGA). Briefly, 5–10 mg of freeze-dried hydrogel bead samples were accurately weighed and placed into a crucible. The samples were heated from 30 °C to 600 °C at a heating rate of 20 °C/min under a programmed temperature control procedure. The weight loss of the hydrogel beads was continuously monitored as a function of temperature and time to evaluate their thermal stability [45].

### 4.7. Structural Characterization of ZJPA-cAMP/CS-SA Hydrogel Beads

#### 4.7.1. X-Ray Diffraction (XRD) Analysis

X-ray diffraction (XRD) measurement was performed by using an X-ray diffraction instrument (Ultima IV, Rigaku, Japan) to test the freeze-dried hydrogel bead powder from hydrogel beads at a scanning rate of 4°/min with the 2θ range from 10 to 60 [46].

#### 4.7.2. Fourier Transform Infrared (FTIR) Spectroscopy Analysis

The chemical structures of the freeze-dried ZJPA-cAMP/CS-SA hydrogel beads were characterized using Fourier transform infrared (FTIR) spectroscopy. Briefly, a small amount of freeze-dried hydrogel bead powder was thoroughly mixed and ground with potassium bromide (KBr), followed by compression into transparent pellets under dry conditions. The prepared pellets were placed in the infrared spectrometer chamber for scanning. The FTIR spectra were recorded in the range of 4000–400 cm^−1^ with a spectral resolution of 1 cm^−1^, and 64 scans were accumulated for each sample [47].

#### 4.7.3. Scanning Electron Microscopy (SEM) Analysis

The microstructure of the freeze-dried ZJPA-cAMP/CS-SA hydrogel beads was observed according to the method reported by Wang et al. [48] with slight modifications. Briefly, a conductive adhesive tape was attached to the sample holder, and the freeze-dried hydrogel beads were evenly dispersed onto the tape surface. The samples were subsequently coated with a thin layer of gold and examined using a scanning electron microscope. The SEM images were obtained at an accelerating voltage of 15 kV under appropriate magnification conditions to evaluate the surface morphology and microstructure of the hydrogel beads.

### 4.8. In Vitro Simulated Digestion

#### 4.8.1. Release in Simulated Salivary Fluid (SSF)

The in vitro simulated salivary digestion was performed according to the method reported by Yao et al. [49] with slight modifications. Briefly, simulated salivary fluid (SSF) was prepared by dissolving NaCl (0.24 mg/mL), KCl (0.30 mg/mL), and lysozyme (0.01%) in 50.0 mL of sterile ultrapure water. A specific amount of hydrogel beads (1 g) was added to 10 mL of SSF (solid-to-liquid ratio of 1:10) and incubated in a shaking incubator at 37 °C and 50 rpm for 2 min. At the end of the incubation, the mixture was centrifuged (8000 rpm, 10 min), and the supernatant was collected to determine the released cAMP. After washing three times with sterile saline, the amount of released cAMP from the hydrogel beads was determined according to the MEY determination method described in Section 4.2.7. The cAMP release rate was calculated using the following Equation (7):

(8) $$\mathrm{Release}\;\mathrm{rate}=\left(W_{1}/W_{2}\right)\times 100\%$$

where W_1_ represents the amount of released cAMP (g) and W_2_ represents the total amount of cAMP initially present in the hydrogel beads (g).

#### 4.8.2. Release in Simulated Gastric Fluid (SGF)

The in vitro simulated gastric digestion was performed according to the method reported by Zhou et al. [50] with slight modifications. The beads remaining from the salivary phase were transferred to 9 mL of SGF (pH 2.0). Then, simulated gastric fluid (SGF) was prepared by dissolving pepsin (3.0 mg/mL), NaCl (0.24 mg/mL), and KCl (0.30 mg/mL) in sterile ultrapure water. The pH was adjusted to 2.0 using 1 mol/L HCl, and the final volume was adjusted to 50 mL. Briefly, 1 g of hydrogel beads after SSF treatment was transferred into 9 mL of SGF and incubated at 37 °C for 2 h under shaking conditions (100 rpm). Sampling was conducted at 30, 60, 90, and 120 min. A 1.0 mL measure of digestive fluid was removed, and then 1.0 mL of fresh, preheated SGF was added to maintain a constant volume. The release rate of cAMP from the hydrogel beads was subsequently determined according to the MEY measurement method described in Section 4.2.7.

#### 4.8.3. Release in Simulated Intestinal Fluid (SIF)

The in vitro simulated intestinal digestion was conducted according to the method reported by Jang et al. [51] with slight modifications. After SGF digestion, the pH of the digestion system was adjusted to 7.0 using saturated NaHCO_3_ solution. Subsequently, trypsin (1 mg/mL) and bile salts (3 mg/mL) were added for simulated intestinal digestion. The samples were incubated at 37 °C for 2 h under shaking conditions (100 rpm), with sampling at 150, 180, 210, and 240 min. A 1.0 mL measure of digestive fluid was removed, and then 1.0 mL of fresh, preheated SIF was added to maintain a constant volume, and the amount of released cAMP was determined according to the MEY measurement method described in Section 4.2.7.

### 4.9. Statistical Analysis

All experiments were conducted with three independent replicate groups (*n* = 3). An independent replicate group was defined as a complete and independent repetition of the entire experimental procedure starting from the preparation of a new batch of hydrogel beads, ensuring the reproducibility of the preparation process and the reliability of the results. Experimental results are expressed as “mean ± standard deviation (SD).” Statistical analysis was performed using SPSS software (version 27.0.1). For single-factor experiments and comparisons between multiple groups, one-way ANOVA was employed to determine whether there were statistically significant differences among the group means and to identify which specific groups differed from each other. All statistical tests used a significance level of *p* < 0.05. For experiments optimized using the response surface methodology (RSM), the experimental data were analyzed using Design-Expert 13 software; a second-order polynomial regression model was fitted to the data, and analysis of variance (ANOVA) was used to assess the statistical significance of this model and its individual terms.

## Figures and Tables

**Figure 1:**
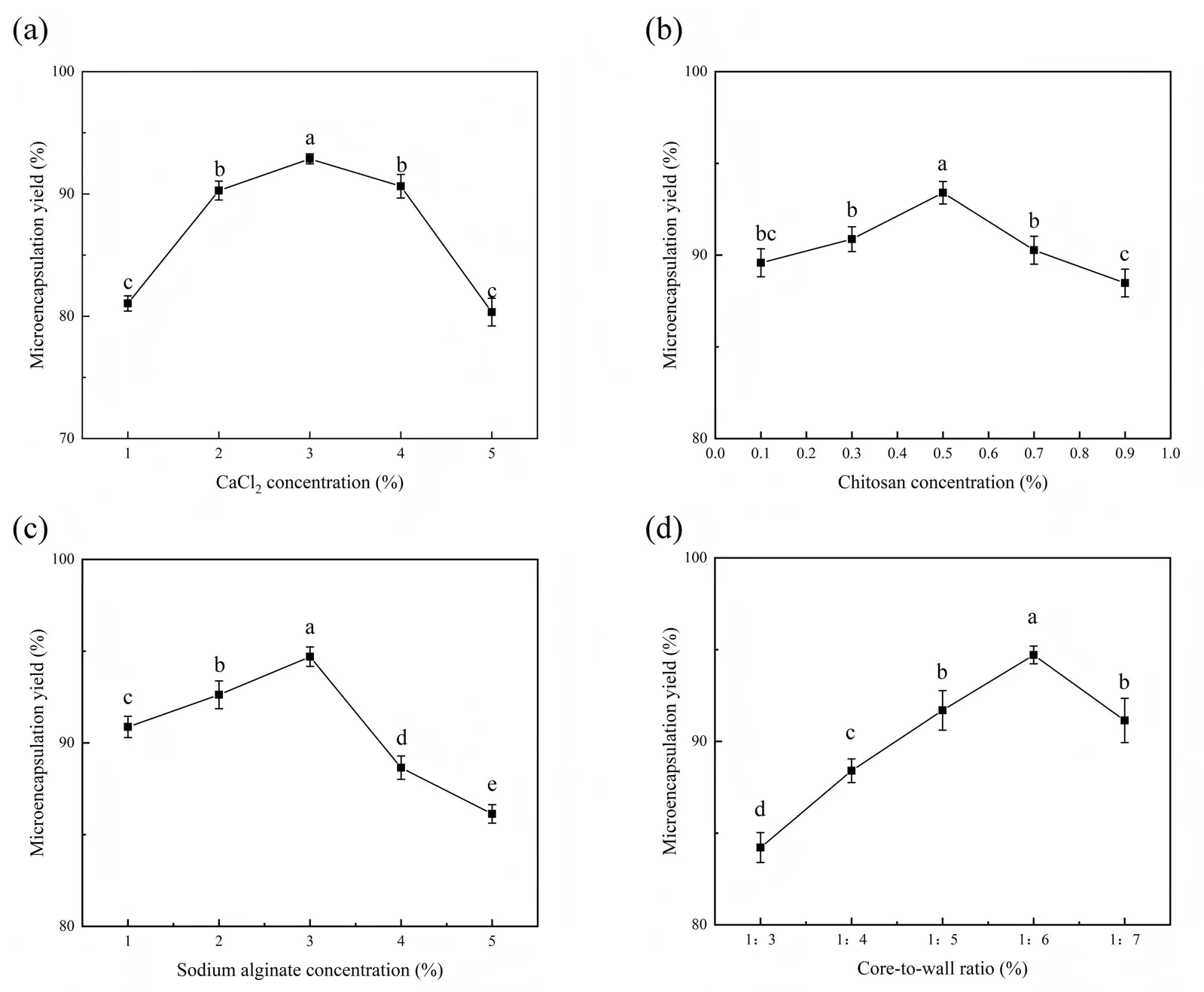
Effects of preparation parameters on the microencapsulation yield (MEY) of ZJPA-cAMP/CS-SA hydrogel beads. The effects of CaCl_2_ concentration (**a**), CS concentration (**b**), SA concentration (**c**), and core-to-wall ratio (g:g) (**d**) on the MEY of hydrogel beads were investigated. All experiments were performed in triplicate (*n* = 3). Different letters indicate significant differences among groups (*p* < 0.05), and vertical error bars represent standard deviations.

**Figure 2:**
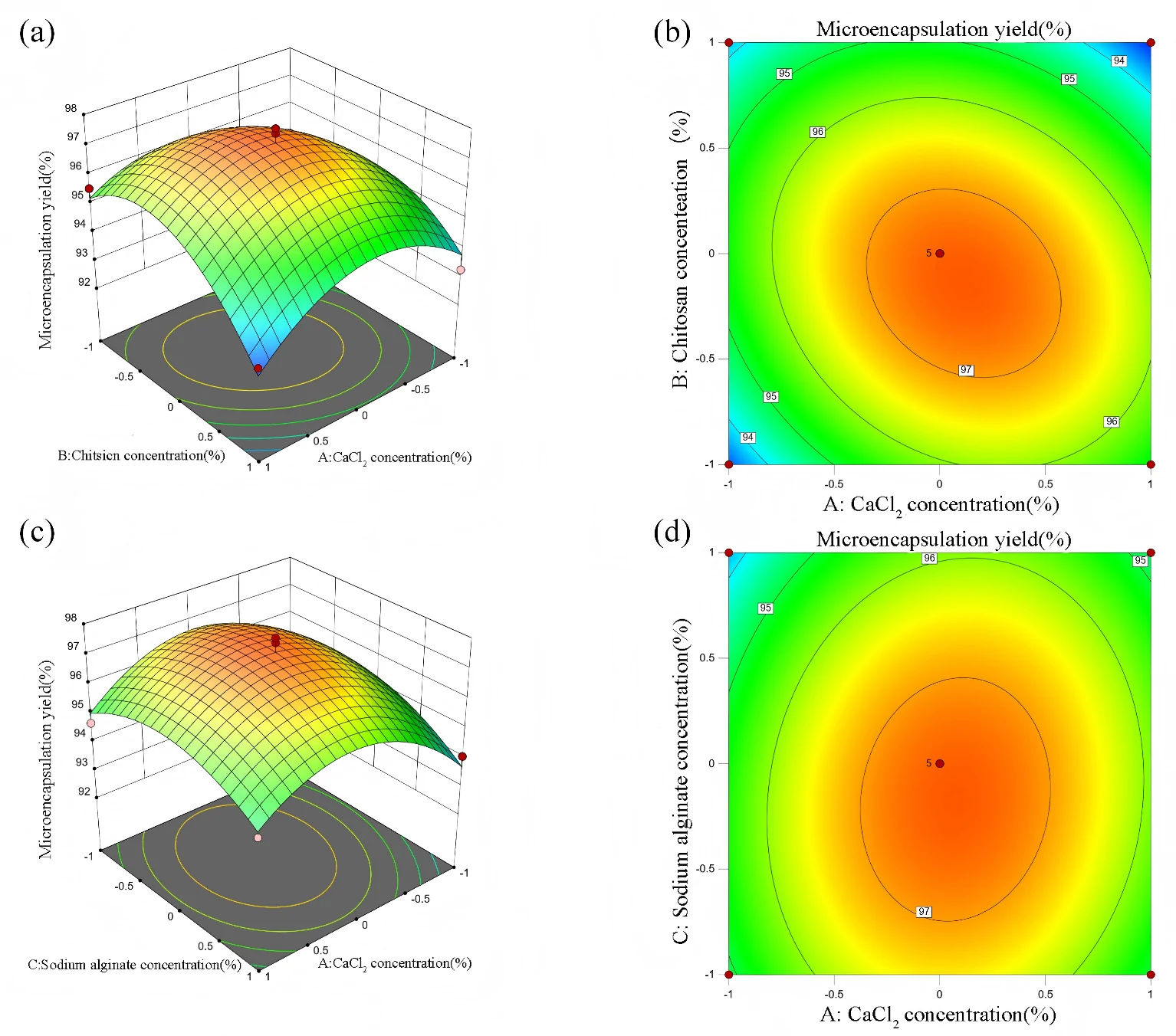

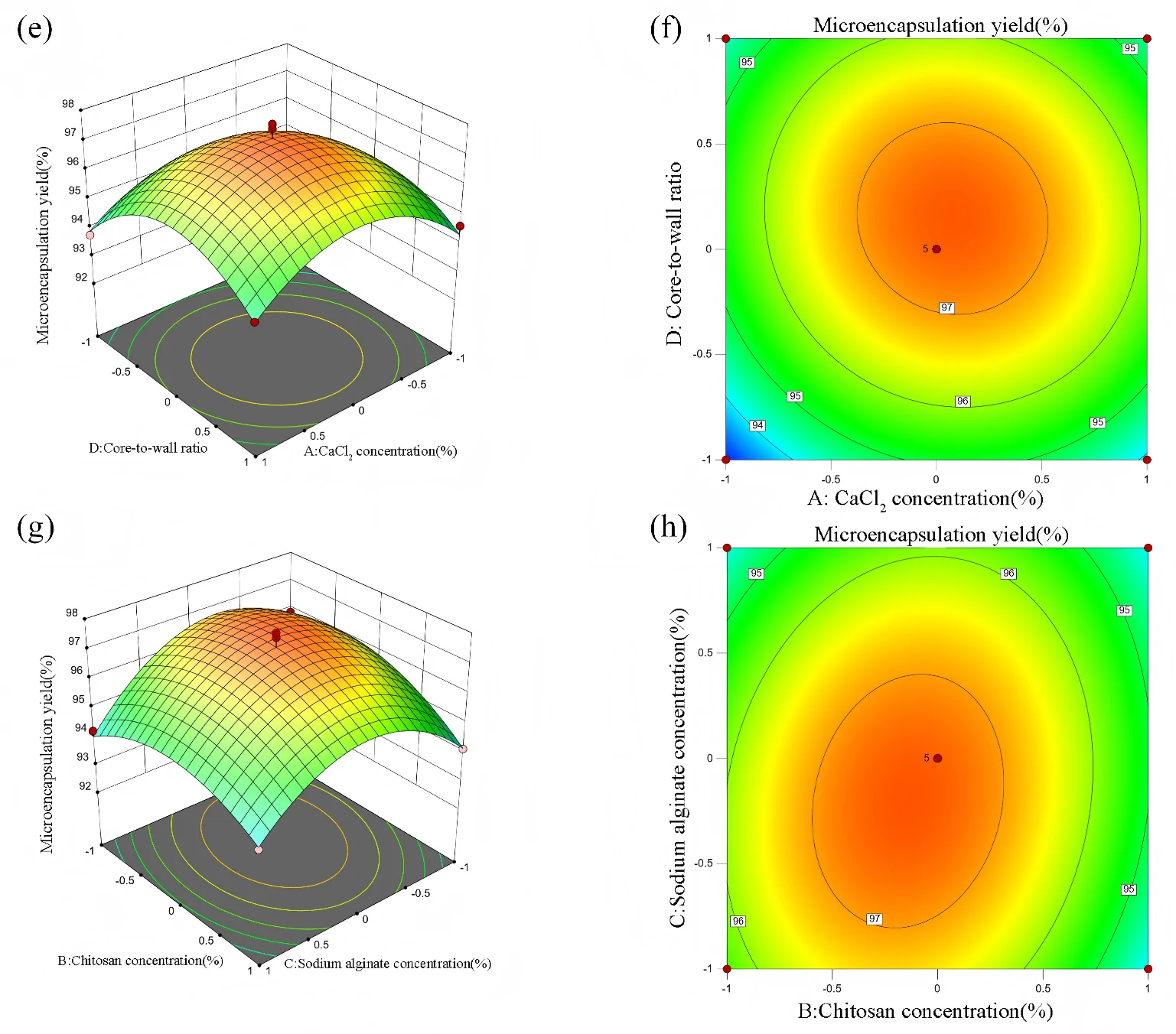

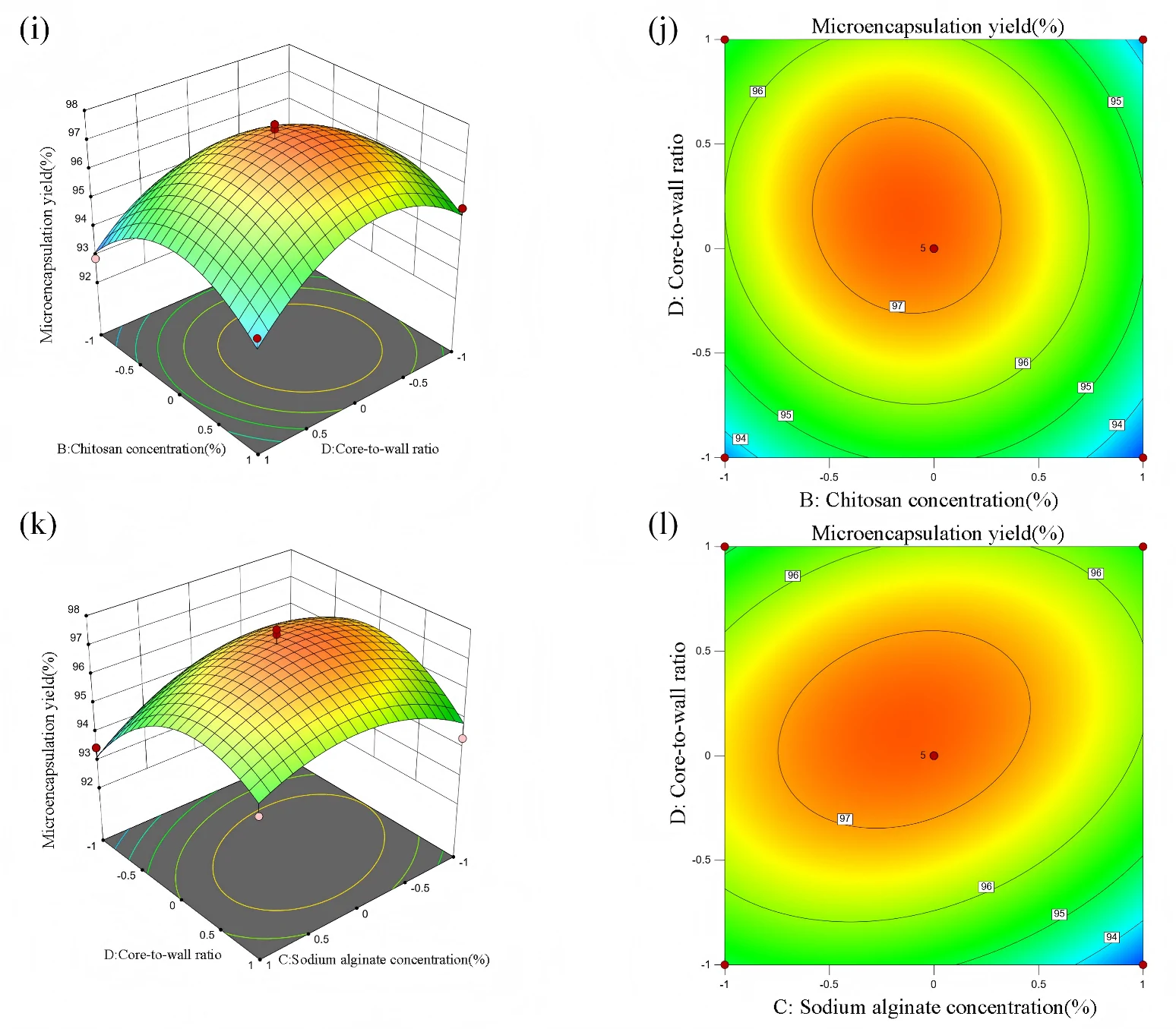
Response surface plots (**a**,**c**,**e**,**g**,**i**,**k**) and contour plots (**b**,**d**,**f**,**h**,**j**,**l**) illustrating the interaction effects of key process parameters on the microencapsulation yield (MEY) of ZJPA-cAMP/CS-SA hydrogel beads. (A–B) CaCl_2_ concentration × CS concentration; (A–C) CaCl_2_ concentration × SA concentration; (A–D) CaCl_2_ concentration × core-to-wall ratio; (B–C) CS concentration × SA concentration; (B–D) CS concentration × core-to-wall ratio; (C–D) SA concentration × core-to-wall ratio.

**Figure 3:**
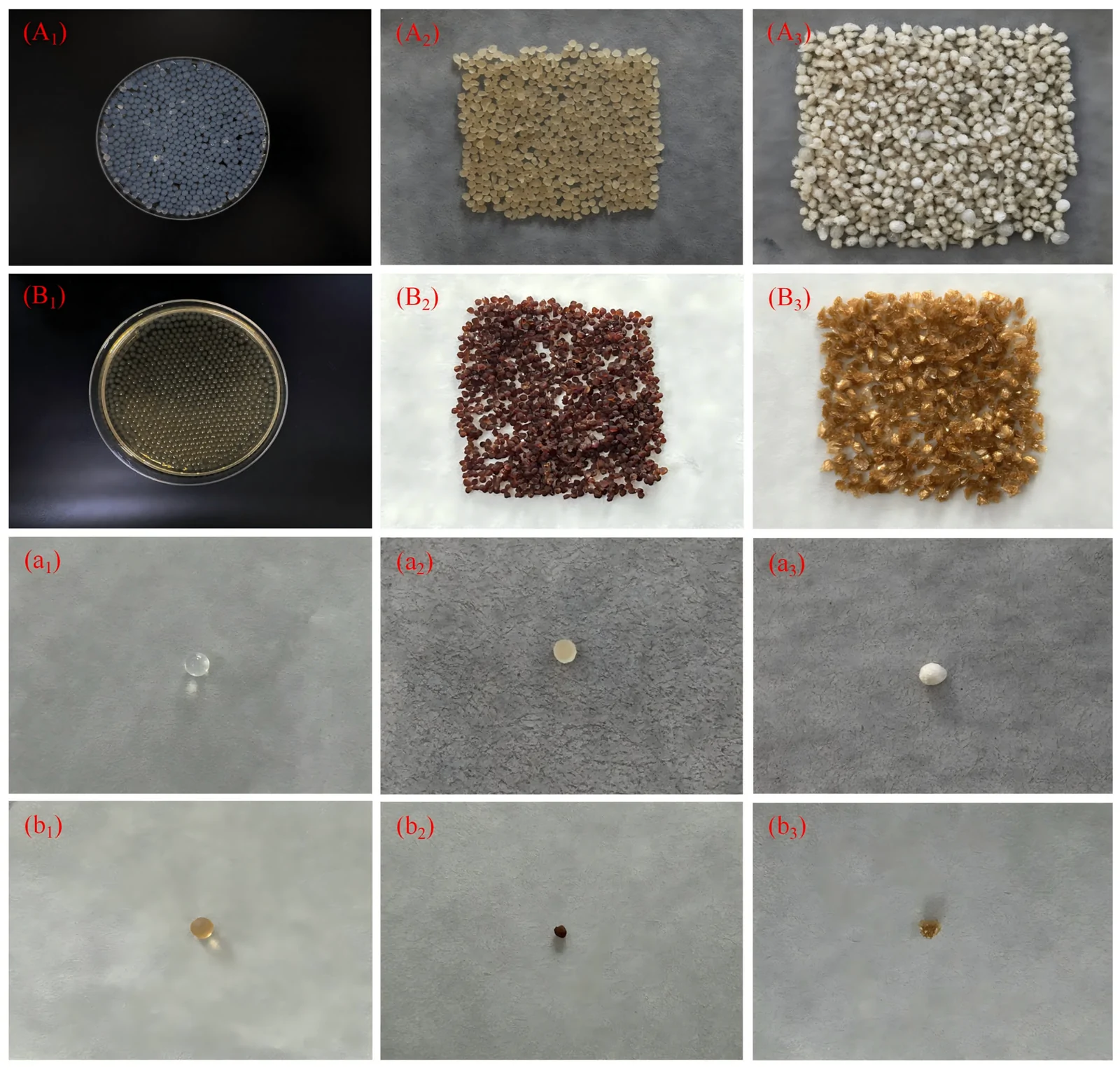
Surface morphology of blank hydrogel beads (**A_1_,A_2_,A_3_,a_1_,a_2_,a_3_**) and ZJPA-cAMP/CS-SA hydrogel beads (**B_1_,B_2_,B_3_**,**b_1_,b_2_,b_3_**) under different drying conditions: fresh state (**A_1_**,**a_1_**,**B_1_**,**b_1_**), oven-dried at 30 °C (**A_2_**,**a_2_**,**B_2_**,**b_2_**), and freeze-dried state (**A_3_**,**a_3_**,**B_3_**,**b_3_**).

**Figure 4:**
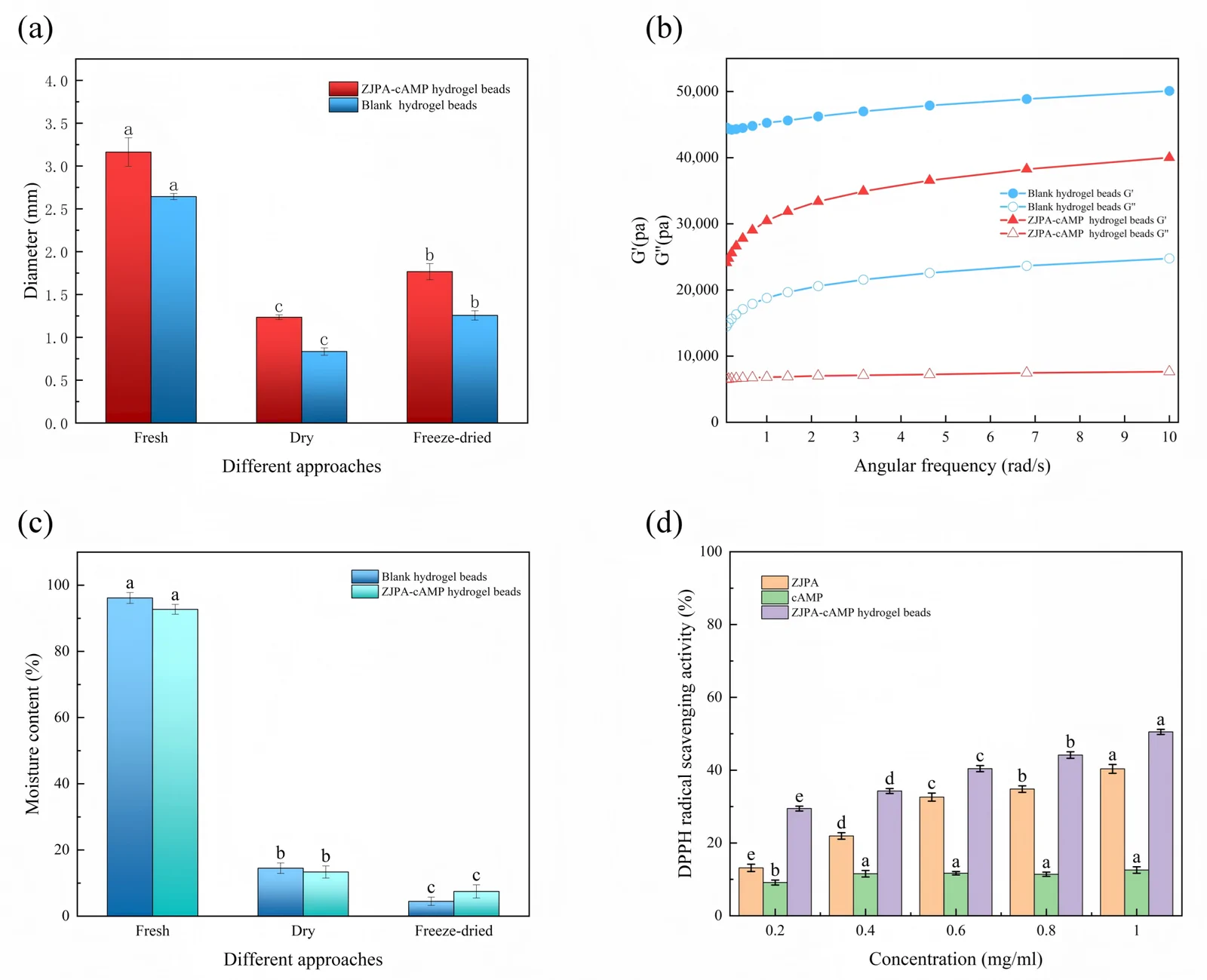
Diameter (**a**), dynamic frequency sweep (**b**), moisture content under different conditions (**c**), and DPPH radical scavenging activity (**d**) of blank hydrogel beads, ZJPA-cAMP/CS-SA hydrogel beads, ZJPA, and cAMP. Different lowercase letters indicate significant differences among groups (*p* < 0.05), and vertical error bars represent standard deviations.

**Figure 5:**
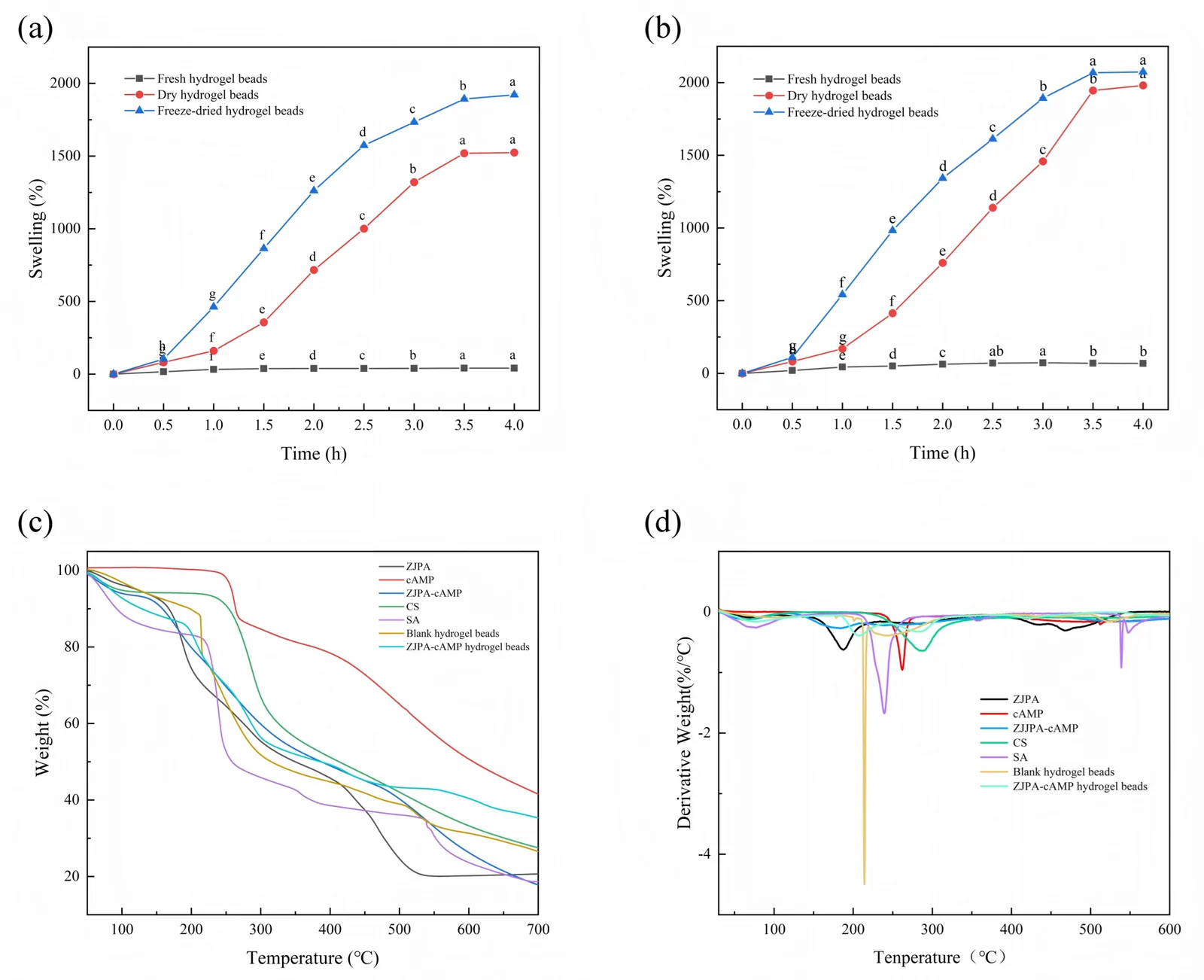
Swelling degree of hydrogel beads under simulated gastric fluid (pH 3.0) and simulated intestinal fluid (pH 7.0) conditions (**a**,**b**); thermogravimetric analysis (TGA) curves (**c**) and derivative thermogravimetric (DTG) curves (**d**) of ZJPA, cAMP, ZJPA-cAMP, CS, SA, blank hydrogel beads, and ZJPA-cAMP/CS-SA hydrogel beads. Different lowercase letters indicate significant differences among groups (*p* < 0.05).

**Figure 6:**
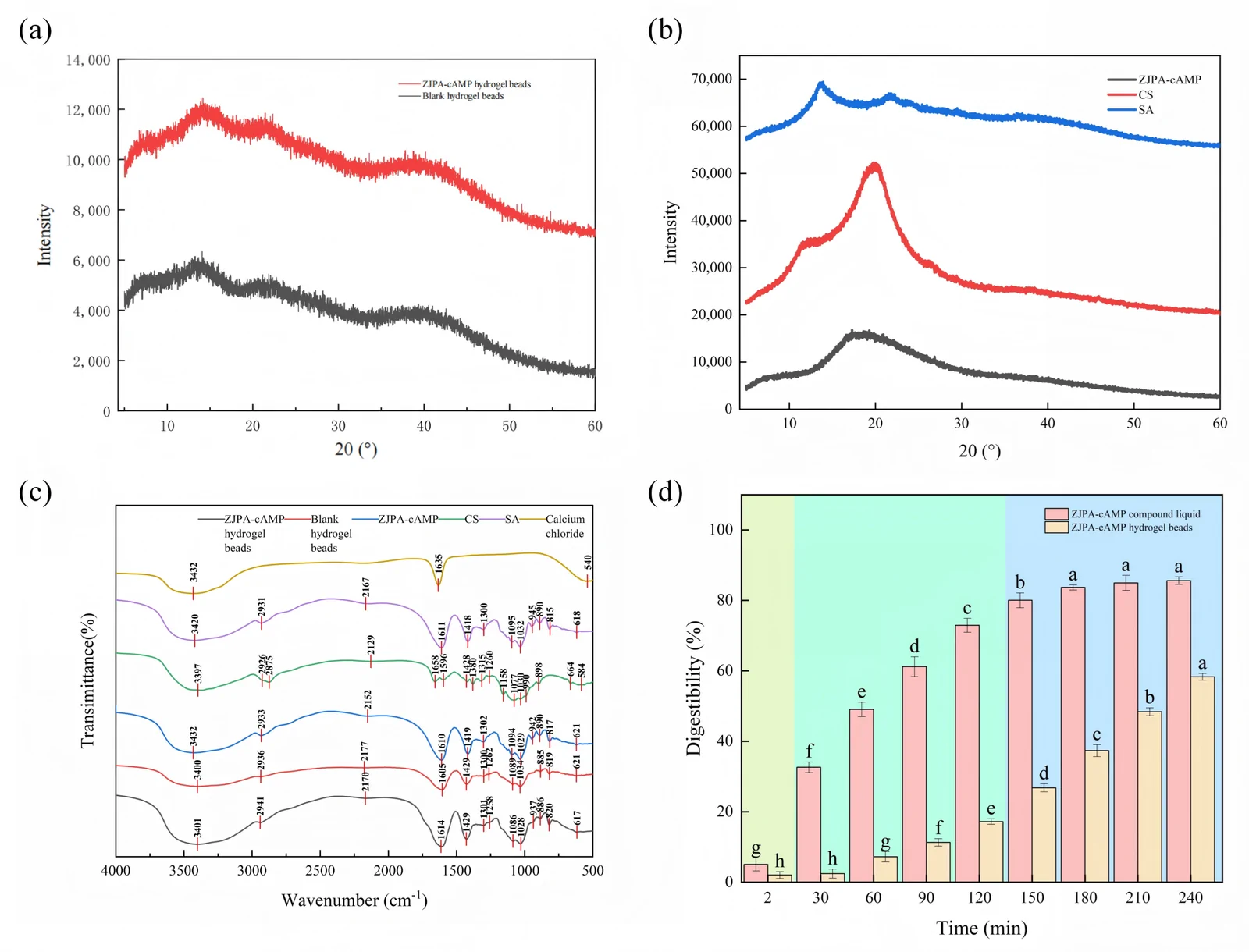
X-ray diffraction (XRD) patterns of ZJPA-cAMP-loaded hydrogel beads and blank hydrogel beads (**a**); XRD patterns of ZJPA-cAMP, CS, and SA (**b**); Fourier transform infrared (FTIR) spectra of ZJPA-cAMP-loaded hydrogel beads, blank hydrogel beads, ZJPA-cAMP, CS, SA, and CaCl_2_ (**c**); in vitro simulated gastrointestinal digestion profiles of ZJPA-cAMP composite liquid and ZJPA-cAMP-loaded hydrogel beads (**d**). The digestion process included the simulated oral phase (0–2 min), simulated gastric phase (3–120 min), and simulated intestinal phase (121–240 min). Different lowercase letters indicate significant differences among groups (*p* < 0.05), and vertical error bars represent standard deviations.

**Figure 7:**
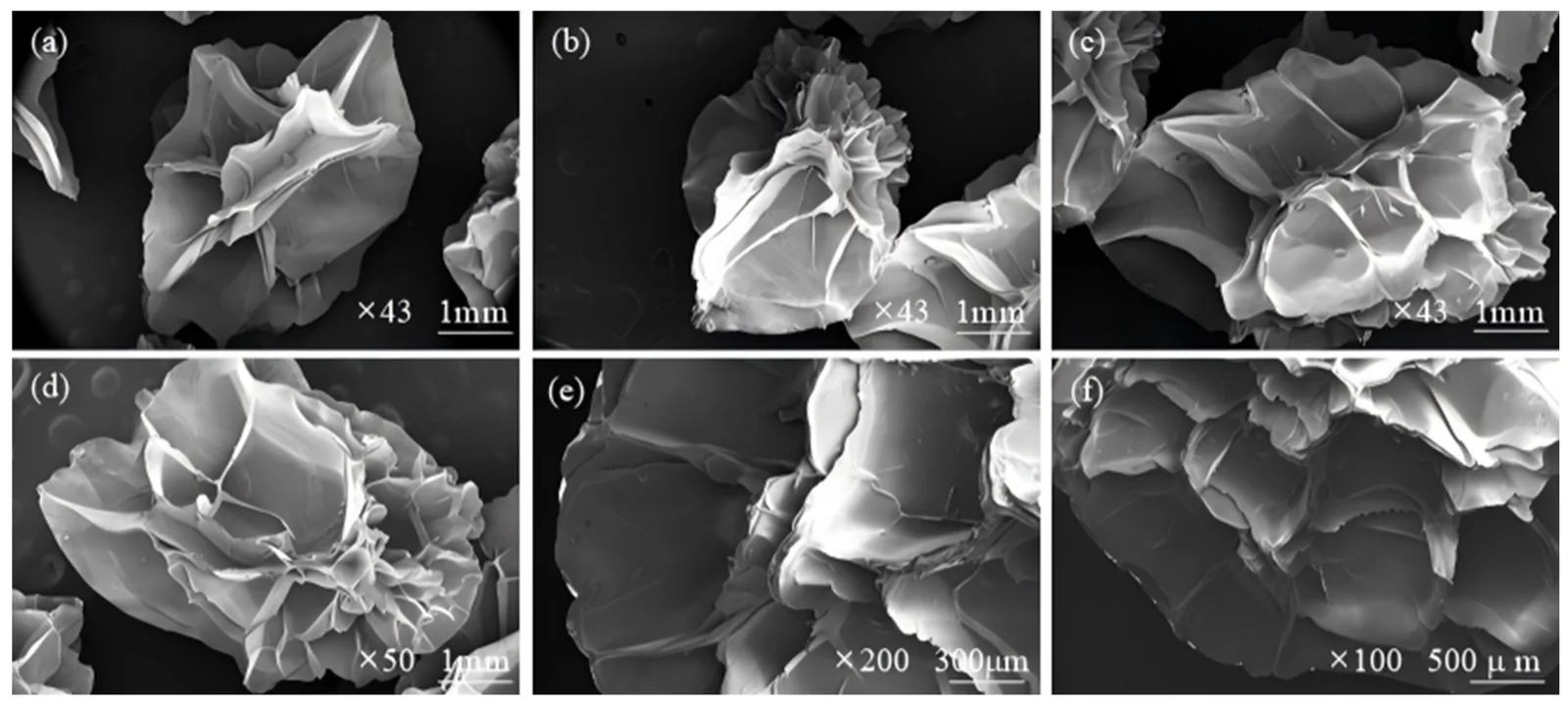
SEM images of freeze-dried ZJPA-cAMP-loaded hydrogel beads at different magnifications; panels (**a**–**c**) are at 43× magnification; panel (**d**) is at 50× magnification; panel (**e**) is at 200× magnification; and panel (**f**) is at 100× magnification.

**Figure 8:**
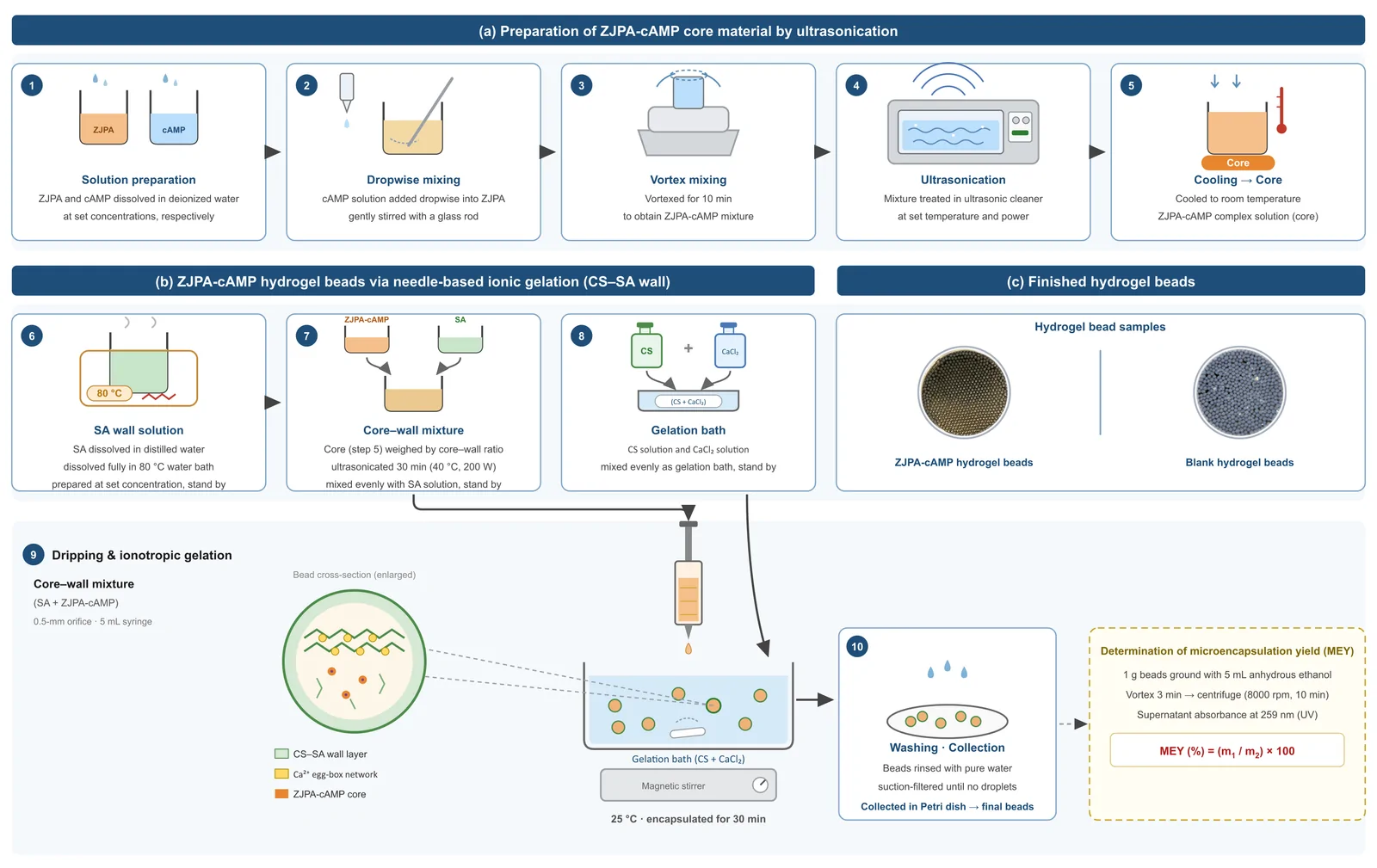
Schematic illustration of the orifice method for the preparation of ZJPA-cAMP/CS-SA hydrogel beads.

**Table 1:** Experimental design and results of the response surface methodology.

Run	A	B	C	D	Microencapsulation Yield %
1	−1	−1	0	0	92.8
2	1	−1	0	0	95.54
3	−1	1	0	0	93.25
4	1	1	0	0	93.23
5	0	0	−1	−1	95.25
6	0	0	1	−1	93.47
7	0	0	−1	1	94.35
8	0	0	1	1	94.89
9	−1	0	0	−1	93.06
10	1	0	0	−1	93.76
11	−1	0	0	1	94.62
12	1	0	0	1	94.58
13	0	−1	−1	0	95.87
14	0	1	−1	0	94.21
15	0	−1	1	0	94.13
16	0	0	0	0	97.21
17	0	1	1	0	93.97
18	−1	0	−1	0	95.08
19	0	0	0	0	96.86
20	1	0	−1	0	94.67
21	−1	0	1	0	94.05
22	0	0	0	0	97.61
23	1	0	1	0	94.53
24	0	−1	0	−1	93.17
25	0	0	0	0	97.05
26	0	1	0	−1	92.88
27	0	0	0	0	97.77
28	0	−1	0	1	95.21
29	0	1	0	1	94.01

**Table 2:** Analysis of variance (ANOVA) for the regression model.

Source	Sum of Squares	df	Mean Square	F-Value	*p*-Value	Significance
Model	55.24	14	3.95	20.29	<0.0001	**
A	0.9919	1	0.9919	5.10	0.0404	*
B	2.23	1	2.23	11.45	0.0045	**
C	1.61	1	1.61	8.26	0.0123	*
D	3.07	1	3.07	15.79	0.0014	**
AB	1.90	1	1.90	9.79	0.0074	**
AC	0.1980	1	0.1980	1.02	0.3301	
AD	0.1369	1	0.1369	0.7039	0.4156	
BC	0.5625	1	0.5625	2.89	0.1111	
BD	0.2070	1	0.2070	1.06	0.3197	
CD	1.35	1	1.35	6.92	0.0198	*
A^2^	18.63	1	18.63	95.77	<0.0001	**
B^2^	21.18	1	21.18	108.91	<0.0001	**
C^2^	6.91	1	6.91	35.52	<0.0001	**
D^2^	18.41	1	18.41	94.64	<0.0001	**
Residual	2.72	14	0.1945			
Lack of fit	2.14	10	0.2142	1.47	0.3777	
Pure error	0.5812	4	0.1453			
Total	57.96	28				
R^2^ = 0.9530	Adjusted R^2^ = 0.9060		Predicted R^2^ = 0.7715			
CV%	0.4656					

Note: ** indicates extremely significant difference (*p* < 0.01); * indicates significant difference (*p* < 0.05).

**Table 3:** Main physicochemical properties of ZJPA-cAMP/CS-SA hydrogel beads.

Water-Holding Capacity (%)	Angle of Repose (°)	Bulk Density (g/cm^3^)	Solubility (%)
72.93 ± 3.93	33.02 ± 1.99	0.60 ± 0.02	38.16 ± 0.75

Note: All data are expressed as mean ± standard deviation (*n* = 3).

**Table 4:** Color characteristics of ZJPA-cAMP hydrogel beads under different conditions.

Hydrogel Bead State	L*	a*	b*	ΔE*
Fresh hydrogel beads	34.17 ± 1.74 ^a^	4.4 ± 0.46 ^a^	26.19 ± 1.78 ^a^	-
Freeze-dried hydrogel beads	28.64 ± 1.17 ^b^	5.56 ± 0.04 ^a^	15.55 ± 0.61 ^b^	12.05
Oven-dried hydrogel beads at 30 °C	25.77 ± 1.16 ^b^	12.66 ± 1.14 ^b^	2.5 ± 0.65 ^c^	26.45

Note: All data are expressed as mean ± standard deviation (*n* = 3). Different lowercase letters within the same column indicate significant differences (*p* < 0.05), whereas the same letters indicate no significant differences.

**Table 5:** Textural properties of blank hydrogel beads and ZJPA-cAMP/CS-SA hydrogel beads.

Hydrogel Bead Sample	Hardness (N)	Cohesiveness	Springiness (mm)	Gumminess (N)	Chewiness (mj)
ZJPA-cAMP hydrogel beads	6.22 ± 3.56 a	0.53 ± 0.1 a	0.47 ± 0.02 a	3.76 ± 2.6 a	1.79 ± 1.29 a
Blank hydrogel beads	7.42 ± 2.39 a	0.56 ± 0.1 a	0.71 ± 0.04 b	4.55 ± 2.21 a	3.29 ± 1.72 b

Note: All data are expressed as mean ± standard deviation (*n* = 3). Different lowercase letters within the same column indicate significant differences (*p* < 0.05), whereas the same letters indicate no significant differences.

**Table 6:** Experimental factors and levels for the response surface methodology (RSM) design.

Level	Factor			
	A: Calcium Chloride Concentration (%)	B: Chitosan Concentration (%)	C: Sodium Alginate Concentration (%)	D: Core-Wall Ratio
−1	2	0.3	2	1:7
0	3	0.5	3	1:6
1	4	0.7	4	1:5

## Data Availability

The raw data generated and analyzed during this study are not publicly available due to project access permissions and privacy regulations. However, the relevant data may be obtained from the corresponding author upon reasonable request.

## Abbreviations

cAMP Cyclic	adenosine monophosphate
ZJPA Jujube	polysaccharides
CS	Chitosan
SA	Sodium alginate
RSM	Response surface methodology
MEY	Microencapsulation yield
FTIR	Fourier-transform infrared spectroscopy
XRD X-ray	diffraction
SEM Scanning	electron microscopy
DPPH	2,2-Diphenyl-1-picrylhydrazyl radical
TGA	Thermogravimetric analysis
WHC	Water-holding capacity
SD	Swelling degree
SSF	Simulated salivary fluid
SGF	Simulated gastric fluid
SIF	Simulated intestinal fluid
ANOVA	Analysis of variance
BBD	Box–Behnken design
